# Harnessing endogenous CRISPR-Cas9 for inducible genetic engineering of *Apilactobacillus kunkeei*

**DOI:** 10.1128/aem.00728-26

**Published:** 2026-07-13

**Authors:** Mahesh S. Iyer, Erik Hagström, Kristina Näslund, Siv G. E. Andersson

**Affiliations:** 1 Molecular Evolution, Department of Cell and Molecular Biology, Science for Life Laboratory, Biomedical Centre, Uppsala University 206112, Uppsala, Sweden; Norwegian University of Life Sciences, Ås, Norway

**Keywords:** honeybees, *Apilactobacillus kunkeei*, lactic acid bacteria, genetic engineering, CRISPR

## Abstract

Despite substantial advances in bacterial genome engineering, functional genetic analysis remains challenging in many non-model bacterial species, particularly among host-associated gram-positive bacteria. The fructophilic species *Apilactobacillus kunkeei* has been investigated for more than two decades and is a dominant member of the honeybee microbiome, where it contributes to pathogen resistance and colony fitness. Nevertheless, the mechanistic investigation of this ecologically important species has remained limited despite its growing probiotic relevance. To enable functional genomics in this organism, we developed an inducible genome-engineering platform that leverages its endogenous Type II-A CRISPR-Cas9 system. The system uses a sakacin-responsive dual-plasmid initiator–effector design in which phage-derived recombineering genes and a single-guide RNA are coordinately expressed, while DNA cleavage is mediated by natively expressed Cas9. Using this approach, we achieved scarless deletion of individual genes, including targets as large as ~25 kb, gene replacement with a fluorescent reporter, C-terminal epitope tagging, and precise nucleotide substitutions, with editing efficiencies approaching 100%. Both plasmids can be readily cured following modification, allowing recovery of clean mutant genotypes. We further demonstrate that endogenous Cas9 can be repurposed for CRISPR interference using a single, self-contained plasmid to enable targeted transcriptional repression. Together, this work establishes a robust strategy for genetic manipulation of *A. kunkeei* and expands the toolkit available for harnessing endogenous CRISPR-Cas systems in genetically recalcitrant, non-model gram-positive bacteria.

**IMPORTANCE:**

Many ecologically and industrially important bacteria remain genetically recalcitrant, limiting functional genomic studies. As research increasingly extends beyond traditional model organisms, these limitations are especially apparent in non-model gram-positive bacteria from host-associated or environmental niches. Here, we establish an inducible genome-editing framework exploiting the endogenous Cas9 system of *Apilactobacillus kunkeei*, a key member of the honeybee microbiota. This approach enables reliable scarless gene deletions, precise nucleotide changes, large-scale genome modifications, and programmable transcriptional repression. By enabling genetic manipulation in *A. kunkeei*, this work facilitates experimental studies of its roles in honeybee health, microbial interactions, and host-associated adaptation, and highlights the potential of endogenous CRISPR-Cas systems for expanding genetic access in non-model bacteria.

## INTRODUCTION

Lactic acid bacteria are widespread members of host-associated and environmental microbial communities and are valued for their contributions to nutrient metabolism, antimicrobial peptides, and probiotic function, making them attractive targets for genetic and functional studies (1–14). CRISPR (Clustered Regularly Interspaced Short Palindromic Repeats) and Cas (CRISPR-associated) gene-based technologies have revolutionized genome engineering by enabling precise and efficient DNA modification (15–31). Notably, endogenous CRISPR-Cas systems are common across lactobacilli, including active Type II-A systems in approximately 30% of genomes (15, 18). Genome engineering in non-model organisms has largely relied on heterologous CRISPR-Cas systems (4–11), often requiring host-specific optimization to mitigate Cas9 toxicity. Harnessing untapped native CRISPR-Cas machinery offers additional advantages, including improved host compatibility and reduced plasmid burden, motivating their exploration in non-model species (32).

Genome engineering approaches have been established in lactobacilli using CRISPR-Cas systems. In *Lactobacillus reuteri*, Cas9-mediated counter-selection combined with RecT-assisted ssDNA recombineering enabled efficient chromosomal deletions and genome modifications (5, 6). Similar Cas9/RecT-based approaches were subsequently applied in *Lactobacillus plantarum*, where editing efficiencies were found to depend on strain background and repair template delivery strategy, and were later improved through optimization of recombineering functions, DNA methylation, and repair template design (7–10). Alternative strategies have employed Cas9 nickase variants in *Lacticaseibacillus casei* (4, 13) and prophage-derived recombineering systems in *L. plantarum* and *Lactobacillus brevis* to facilitate gene deletion and gene replacement (11). In species such as *Lactobacillus acidophilus*, heterologous SpyCas9-based editing portable platforms have also been developed and extended to additional lactobacilli (14). In parallel, several lactobacilli, including *L. casei*, *Lactobacillus rhamnosus*, *Lactobacillus gasseri*, *Lactobacillus jensenii,* and *Lactobacillus pentosus*, have been shown to possess naturally active endogenous Type II CRISPR-Cas systems (15). Endogenous CRISPR-Cas systems have subsequently been repurposed for genome engineering, including the use of the native Type I-E CRISPR-Cas system of *Lactobacillus crispatus* (12) and the endogenous Type II-A CRISPR-Cas9 system of *Lactobacillus paracasei* (13) and *L. gasseri* (15) for targeted genome modification. Together, these studies demonstrate the versatility of both heterologous and endogenous CRISPR-Cas-based genome engineering approaches in lactic acid bacteria while highlighting the diversity of strategies used across species.

The gut microbiome of honeybees is thought to play an important role in maintaining bee health and colony stability, which is critical for the pollination of food crops worldwide, contributing significantly to agricultural economies (33–35). Among these, the fructophilic species *A. kunkeei* is an important inhabitant of the honeybee hive environment and is consistently detected in the honey crop, hive products, flowers, and fruits (36). *A. kunkeei* has been a subject of research for more than two decades, and studies have focused on the structure and evolution of its 1.5 Mb genome (37, 38), heterofermentative metabolism (39), secreted particles (40), small proteins (41), and its potential as a paratransgenic candidate (42) for probiotic evaluation in honeybees. Furthermore, a certain number of *A. kunkeei* strains contain plasmid-encoded gene clusters for the synthesis of bacteriocins/antimicrobial peptides that are active against major honeybee pathogens such as *Melisococcus plutonis* and *Paenibacillus larvae* (38, 43). Beyond its role in honeybees, *A. kunkeei* has also attracted attention for potential applications in human health (44, 45). Despite the growing interest, genetic manipulation of *A. kunkeei* remains challenging. Transformation and heterologous gene expression have been demonstrated in only a limited number of strains, whereas many isolates remain recalcitrant to genetic modification, limiting deeper mechanistic studies (42, 46).

Many non-model gram-positive bacteria remain difficult due to barriers in DNA delivery, inefficient homologous recombination, and instability or toxicity associated with exogenous gene expression. Alternatively, inducible expression systems provide a promising route to mitigate toxicity and to coordinate editing steps. Nisin- and sakacin-responsive promoters, in particular, are widely used for regulated gene expression in lactic acid bacteria and have supported controlled production of heterologous proteins and recombinases in both laboratory and industrial contexts (42, 47–53). However, the integration of inducible regulation, endogenous CRISPR-Cas activity, and recombineering has not been previously reported for *A. kunkeei*.

Here, we developed a dual sakacin-inducible CRISPR-Cas9 platform that exploits the endogenous nuclease activity of *A. kunkeei* while providing regulated expression of sgRNA and recombineering genes. This strategy enables efficient gene deletion, replacement, epitope tagging, and precise nucleotide modification in otherwise genetically recalcitrant strains. In addition, truncated sgRNAs allow repurposing of endogenous Cas9 for CRISPR interference (CRISPRi), expanding the system to programmable transcriptional control. Overall, the availability of a reliable genome-engineering platform will facilitate mechanistic studies of host association, microbial competition, and probiotic function in *A. kunkeei*, thereby enabling functional investigation of traits relevant to honeybee health.

## RESULTS

### Endogenous Type II-A CRISPR-Cas systems are widespread and active in *A. kunkeei*

**Fig 1:**
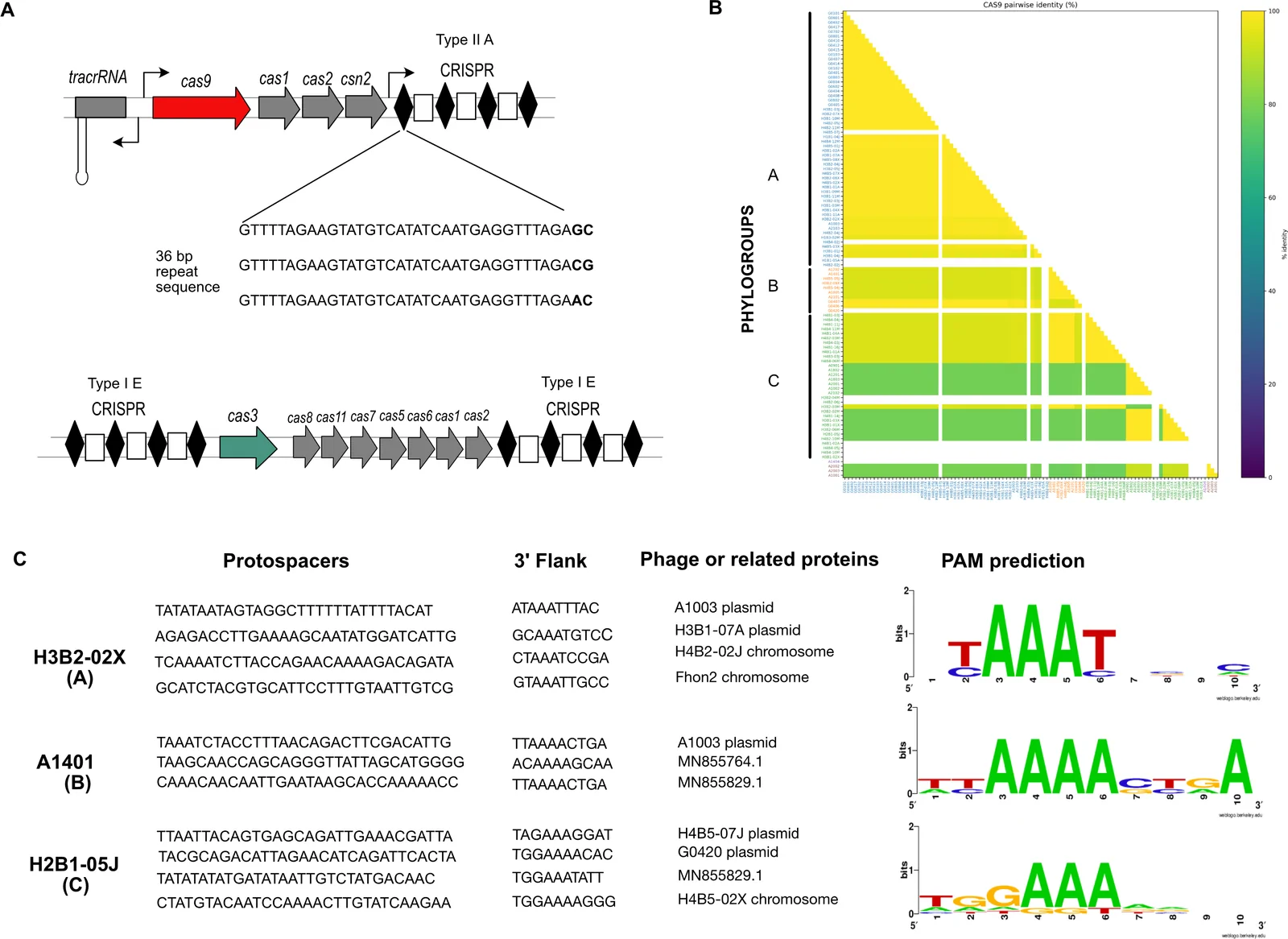
CRISPR-Cas gene clusters in *A. kunkeei* strains. (**A**) Architecture of CRISPR-Cas clusters Type II-A and Type IE identified in *A. kunkeei* strains. The Type-IIA consisted of *cas9*, *cas1*, *cas2,* and *csn2* genes with tracrRNA found upstream of *cas9* (marked in red). Type IE was identified in one strain (H4B5-04J) with *cas3* nuclease (marked in cyan) and *cas* genes. The *cas* genes were followed by repeat (black diamond) and spacer (square box) regions. The 36 bp consensus repeat sequences of Type II-A were identical except at the last two nucleotides for all the strains having the CRISPR-Cas clusters. (**B**) The heatmap shows the lower triangular matrix of pairwise protein sequence identity for Cas9 proteins across 102 *A. kunkeei* strains, plotted according to a predefined phylogroup order (A–F). Strain labels are colored by phylogroup classification: blue (group A), orange (group B), green (group C), purple (group E), and brown (group F). White spaces indicate either the absence of a full-length Cas9 protein or alignment coverage below 50%. The diagonal represents within-strain identity (100%). (**C**) Select protospacers with e value <10^−05^ for each of the representative strains for each phylogroup, H3B2-02X (phylogroup A), A1401 (phylogroup B), and H2B1-05J (phylogroup C) were depicted. Most of the hits of each of the protospacers belonged to either phage or phage-related proteins residing on plasmid or chromosome and were detected within the *A. kunkeei* clade. For Type II-A, 10 nucleotides from the 3′ end of each protospacer were aligned together to generate a Weblogo for protospacer-adjacent motif (PAM) prediction.

To evaluate the feasibility of exploiting natively expressed Cas9 nuclease for genome engineering, we examined its occurrence in 102 strains categorized into six phylogroups (A–F) (38). Using CRISPRCasFinder (54), we identified Type II-A loci in 93 strains (~91% prevalence), of which 3 genomes carried putative disruptive mutations in the *cas9* gene, leaving 90 genomes with intact systems for comparative analysis (Table S1). The loci contained the *cas9*, *cas1*, *cas2*, and *csn2* genes adjacent to repeat–spacer arrays and a predicted tracrRNA upstream of the *cas9* gene in all the strains (Fig. 1A). Within the *A. kunkeei* strains, the amino acid sequence identities of the Cas9 proteins were >94% in isolates assigned to phylogroups A/B. Interestingly, the Cas9 proteins in phylogroup C strains could be sorted into two subtypes, one of which (subtype I) was closely related to the Cas9 proteins in strains of phylogroup A/B, whereas the other (subtype II) was more divergent (<80%) (Fig. 1B; Fig. S1; Table S2). Similar patterns of sequence divergences were also observed for the Cas1, Cas2, and Csn2 proteins (Fig. S1; Table S2).

The crRNA repeats were highly conserved in sequence, differing by at most one nucleotide among strains of different phylogroups (Table S1). We observed consistent lengths of the repeats (36 bp) and spacers (ca 30 bp) in all isolates, albeit with a variable number of spacers ranging from 8 to 52 (phylogroup A strains), 10 to 29 (phylogroup B strains), 9 to 27 (phylogroup C strains), and 22 (phylogroup F strains) (Table S1). Analyses of spacer–protospacer matches identified numerous targets within other *A. kunkeei genomes*, with many hits mapping to genomic regions previously annotated as prophage or phagemid-associated sequences (Table S3). All identified PAMs across all 102 *A. kunkeei* strains shared the extended AAA tract at the 3′ end (Table S4), consistent with other lactic acid bacteria (15). Representative strains from three phylogroups (H3B2-02X of phylogroup A, strain A1401 of phylogroup B, and strain H2B1-05J of subtype II, phylogroup C) revealed 5–6 nt PAMs with distinct sequences (Fig. 1C).

To validate CRISPR-Cas activity, define guide RNA architecture, and predict the crRNA-tracrRNA duplex structures, we performed small RNA sequencing of the three representative strains (Table S5). Mature crRNAs were abundant and displayed precisely processed crRNAs, consisting of 21-nucleotide repeat-derived and 20-nucleotide spacer-derived segments (Fig. S2). However, the read coverage varied extensively among spacers within an array, with the highest coverages noted for the first six spacers (Fig. S2). A similar sharp processing boundary was observed for the tracrRNAs, although with lower read coverage (Fig. S3A). The tracrRNA structures consisted of the five characteristic modules: upper stem, bulge, lower stem, nexus, and GC-rich transcription terminator hairpin, and RNase III cleavage sites (Fig. S2) (55, 56). Predictions for the tracrRNAs were conservative, as a single transcription-terminating hairpin was found to be a part of the final tracrRNAs. We also investigated a leader-derived RNA (ldrRNA) (15) originating from the 5′ end of the CRISPR-Cas array (Fig. S3B). The ldrRNA comprised 20 nucleotides of leader sequence followed by a 21-nucleotide repeat-derived segment, resulting in a transcript architecture analogous to mature crRNAs.

To place these features in a broader context, we compared representative *A. kunkeei* systems with a few previously characterized bacterial Type II CRISPR-Cas systems. *A. kunkeei cas* loci conform to the general architecture of Type II-A CRISPR-Cas systems (Table S7) (27). *A. kunkeei* encodes a large Cas9 protein (~1,400 amino acids), which is highly diverged in amino acid sequence (<50%) compared to its homologs in other species (Fig. S3C). Despite this sequence divergence, comparative domain architecture analysis (57, 58) revealed conservation of the characteristic RuvC, HNH, REC, and PAM-interacting domains across the Cas9 homologs examined (Fig. S3D). Across the compared systems, repeat and spacer lengths were generally conserved at 36 nts and ~30 nts, respectively, whereas repeat sequences, tracrRNA sequences, and PAMs varied substantially (Table S6).

To assess the activity of the endogenous Cas9 nuclease, we performed plasmid interference assays in the representative strains (Fig. S4). Plasmids carrying a cognate protospacer and matching PAM showed a strong reduction in transformation efficiency, whereas control plasmids lacking either element were readily maintained, confirming active *in vivo* targeting by natively expressed Cas9 protein. Strain-specific PAM preferences were observed and were consistent with *in silico* predictions (Table S3). In strain A1401, the strongest interference occurred with ATAAA and GTAAA PAMs, with mutational analysis indicating that positions 2–5 are critical for activity (Fig. S4A). In strain H3B2-02X, GCAAA and ATAAA supported robust interference (Fig. S4B), while in strain H2B1-05J, targeting was observed with (T/A/C)GGAAA PAMs (Fig. S4C). Genome-wide PAM motif analysis revealed that five-nucleotide PAMs occur at frequencies of approximately 3–5 sites per kilobase, whereas six-nucleotide PAMs were less common (Table S7). PAM occurrences varied between coding and noncoding strands in a strain-or PAM-dependent manner (Table S7), consistent with sequence distribution rather than strand-specific Cas9 activity. Together, these features indicate that the endogenous Type II-A system is intact and functional.

### Establishment of sakacin induction for endogenous Cas9 in *A. kunkeei*

Sakacin-inducible promoters have been widely used in lactic acid bacteria as tightly controlled expression systems. To evaluate their utility in these strains, we used a *gusA* reporter plasmid pSIP411 (53) in which *gusA* expression was placed under sakacin control. We monitored β-glucuronidase activity across a concentration range of 0–5 ng/mL (Fig. S5A). All three representative strains showed a clear dose-dependent response, with induction detectable at 1 ng/mL and saturation reached by ≥2 ng/mL (Fig. S5A). *A. kunkeei* strain H2B1-05J displayed markedly higher activity compared to strains A1401 and H3B2-02X. Uninduced controls displayed low background activity, confirming the promoter’s tight regulation.

To enable targeted genome editing in three representative *A. kunkeei* strains, we implemented a sakacin-inducible recombineering system utilizing its endogenous Cas9 nuclease. The system involved expression of a PLE3 phage-derived (12) single-strand annealing recombinase together with exonuclease and gam functions under control of a sakacin promoter (pSH71 origin, chloramphenicol resistance marker), referred to as pMER. As a proof of principle, we targeted the *rpoB* gene with synthetic oligonucleotides (~100 nt, 100 µg) carrying rifampicin resistance mutations. Upon sakacin induction (10 ng/mL), robust and reproducible recovery of rifampicin-resistant colonies was observed in multiple replicates of all three strains, and sequencing confirmed the expected *rpoB* point mutations in most of the tested colonies (Fig. S5B and C). The induced controls without oligonucleotide yielded few colonies or no colonies. Together, these results demonstrate that sakacin-inducible control is both tunable and reliable in *A. kunkeei*, providing a robust regulatory module for subsequent genome engineering.

### Repurpose endogenous Cas9 with initiator–effector plasmid modules for small and large gene scarless deletions

**Fig 2:**
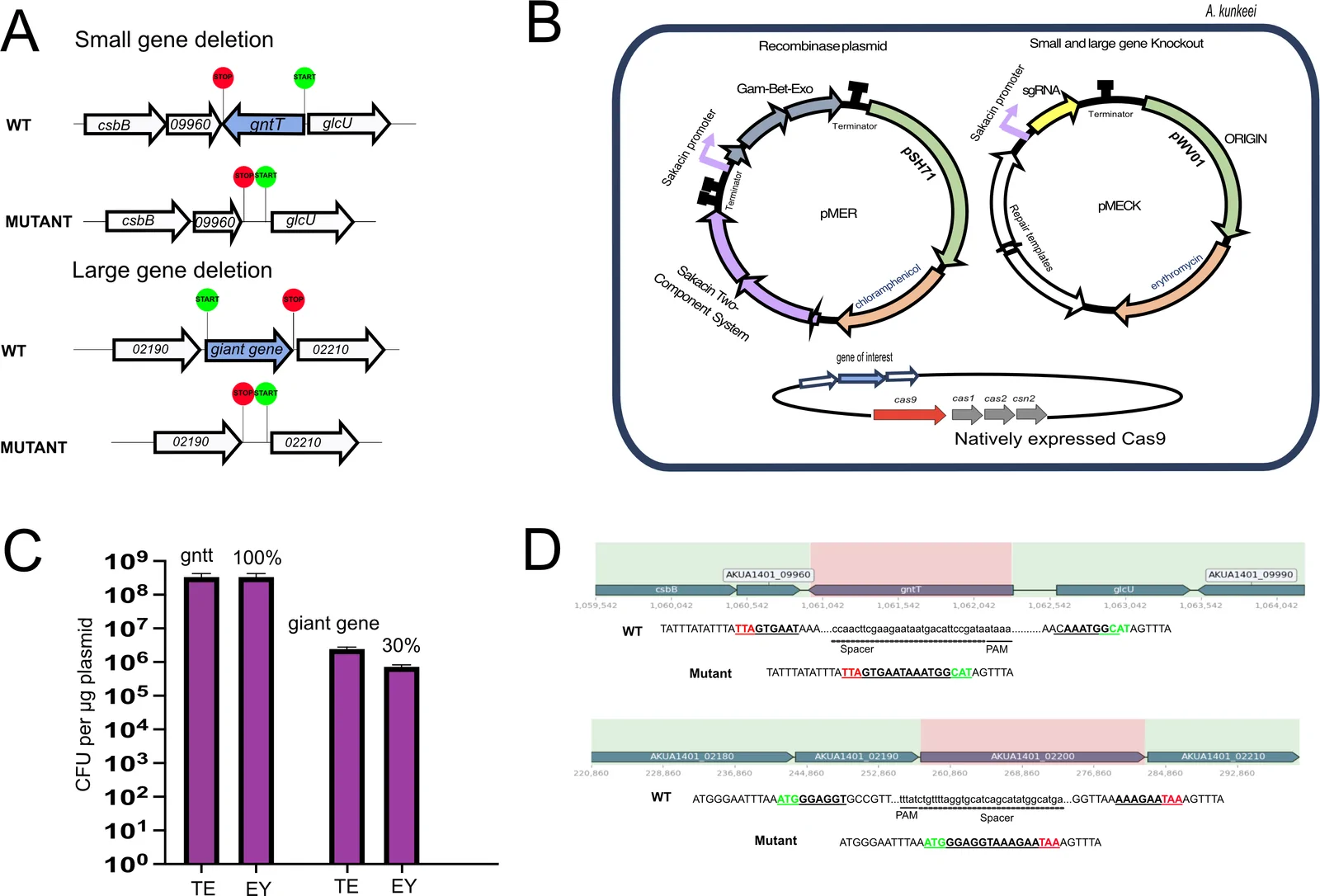
Dual-plasmid endogenous CRISPR-Cas genome editing for gene deletions. (**A**) Schematic depicting the scarless deletion of a small gene (*gntT* 1,356 bp) and a large gene (AKUA1401_02200 25 kb). The target gene is highlighted in blue, whereas the flanking genes are white. The start and stop codons are highlighted as colored circles. (**B**) The pMECK plasmid contains the repair templates and encodes the sgRNA under the control of a sakacin-inducible promoter, enabling programmable targeting of chromosomal loci. The pMER plasmid carries the *gam–bet–exo* recombination machinery and sakacin two-component kinase-regulator system to facilitate homology-directed repair and regulate cognate promoters, respectively. Repair templates are 100 bps upstream of the PAM for the giant gene AKUA1401_2200, whereas around 315 bp upstream of the PAM for the *gntT* gene. Both plasmids are maintained under compatible replication origins (pWV01 and pSH71) and selectable with erythromycin or chloramphenicol markers, respectively. Upon induction with the sakacin signaling peptide, the sgRNA and recombinase genes are co-expressed, resulting in Cas9-mediated double-stranded DNA cleavage at the target locus. The system operates using the endogenous Cas9 machinery that cleaves and counter-selects WT cells and repairs via homology-directed recombination, which enabled precise deletion of the *gntT* gene and large-gene knockout. Nine nucleotides from the start and end of the gene are retained to prevent any downstream effects. (**C**) Editing efficiencies are presented as colony-forming counts per microgram pMECK plasmid and expressed as mean ± standard deviation, where TE represents the apparent transformation efficiency (or final viable colony yield), and EY denotes the editing yield. As editing experiments involve extended recovery and selective enrichment steps, the resulting transformation efficiencies appear higher than true transformation efficiencies. CFU was calculated as the number of colonies obtained per microgram of pMECK plasmid DNA transformed following all recovery, induction, and selection steps, whereas editing yield was determined as the percentage of edited colonies relative to the total number of transformants. (**D**) DNA Feature Viewer visualization of sequenced genomes confirms targeted deletion events at the chromosomal locus, which are highlighted in pink. The nucleotides around the deletion site are presented as text.

Next, we focused detailed editing trials on strain A1401, which consistently yielded higher transformation recoveries compared to the other two strains, demonstrating the full range of genome modifications achievable in *A. kunkeei*. We performed the editing trials under induced conditions with the endogenous Cas9 directed for self-targeting and chromosomal modification. For this purpose, we chose the *gntT* (1356 bp) gene, encoding a membrane-bound gluconate transporter, as the primary target (Fig. 2A). A single-guide RNA (sgRNA) based on the tracrRNA:crRNA duplex with the ATAAA PAM was used for the edits. A 1 kb donor DNA encompassing homology arms flanking the *gntT* gene was designed to facilitate homology-directed repair while retaining the first and last nine nucleotides of the coding sequence to minimize potential downstream effects.

For genetic engineering purposes, we developed a dual-sakacin two-plasmid system, pMER and pMECK (Fig. 2B). These represent an initiator–effector configuration, where pMER (PLE3 recombinase under sakacin-inducible PorfX promoter, sakacin two-component system, pSH71, chloramphenicol resistance marker) initiates recombinase activity and induction control, and pMECK (sgRNA under sakacin-inducible PorfX promoter, repair template plasmid, erythromycin resistance marker, and pWV01 origin) provides the effector components for natively expressed cas9 targeting. It should be noted that genome modifications are generated through homologous recombination mediated by the repair template and recombineering functions, while Cas9 cleavage counter-selects cells retaining the wild-type genotype, thereby enriching for edited cells. Unlike the pMER plasmid having a fixed architecture, the pMECK plasmid is modular between different gene modifications. *A. kunkeei* strain A1401 was transformed with pMER and subsequently with pMECK with exogenous addition of the sakacin peptide. We observed that the editing was highly efficient, as all 16 randomly selected colonies (100%) confirmed by colony PCR carried the intended deletion (Fig. 2C). We obtained a complete scarless gene knockout in *A. kunkeei* using its natively expressed Cas9, further verified by whole-genome sequencing of a plasmid-cured colony (Fig. 2D). Apart from the intended deletion, no additional sequence changes were detected when compared to the parental genome or the pMER-carrying strain, indicating minimal off-target activity.

To further evaluate the capacity of the platform, we targeted a very large gene of ~25 kb (AKUA1401_02200) for deletion (Fig. 2A). Using the same initiator–effector configuration (pMER plus pMECK with the appropriate sgRNA and 1 kb repair template arms) in conjunction with the endogenous cas9 gene (Fig. 2B), we obtained ~30% efficiency among the screened colonies (Fig. 2C), further confirmed by genome sequencing (Fig. 2D). These results show that the native *A. kunkeei* Cas9 enzyme has substantial capability for large-fragment genome editing. Collectively, these results illustrate that the system is applicable to both small-gene and large-gene deletions.

### Design optimization, control experiments for gene deletion, and plasmid curing

To dissect the contributions of recombinase and CRISPR-Cas9 in genome editing, we first tested whether recombinase alone could mediate integration of linear double-stranded DNA with and without phosphorothioate (PT) modified ends (Table S8). Cells carrying pMER were transformed with a linear cassette encoding an erythromycin resistance marker flanked by *gntT* homology arms. Even with 10 µg of DNA, no colonies were recovered. Besides, no colonies were obtained even after using the plasmid-borne repair template.

Next, we tested CRISPR-Cas9 in the absence of pMER. In the wild-type background, introducing pMECK with the *gntT* sgRNA under sakacin control together with a repair template produced many colonies, but none carried the intended deletion, consistent with the lack of sgRNA expression without the two-component regulation (Table S8). pMECK with a repair template alone without sgRNA also yielded colonies, but no cells were modified. Replacing the inducible promoter in pMECK with a constitutive *tuf* promoter led to recovery of five colonies, four of which carried the correct knockout. Control experiments confirmed that the constitutive *tuf* promoter-driven sgRNA without a repair template was lethal, and that repair templates combined with either no sgRNA or a mock sgRNA produced colonies but no modifications. In the pMER carrying background, final viable colony yield greatly increased (~5 vs ~40 without extended induction, and ~50 vs ~310 with an additional day of induction and double antibiotic selection), and all screened colonies contained the correct deletion. Thus, co-expression of recombinase with the Cas9 enzyme markedly enhanced both colony recovery and editing accuracy.

To further optimize knockout design parameters in the presence of pMER, we turned to the sakacin-inducible pMECK construct with repair templates (Table S8). We compared the sgRNAs targeting the coding versus the noncoding strand of *gntT*. The design placing the PAM on the non-coding strand (sgRNA targeting the coding strand) yielded higher recovery and successful edits, whereas the design placing the PAM on the coding strand produced no colonies. For *gntT*, PAMs were predominantly located on the non-coding strand. To assess whether this reflected a gene-specific feature, we next targeted AKUA1401_00910 (1152 bp), located near a putative type II restriction–modification gene, where PAMs were more frequent on the coding strand; in this case, a design with the PAM on the coding strand gave edits with 100% efficiency. Whole-genome sequencing of the cured colony confirmed the results. Taken together, these observations suggest that editing efficiency can depend on locus- and strand-specific contexts, rather than a strand bias of the CRISPR-Cas system.

In parallel, we tested repair templates with homology arms of different lengths (200 bp, 400 bp, 600 bp, 800 bp, and 1 kb per arm) and how that affected editing efficiency in the presence of pMER (Table S8). All lengths under 800 bp per arm failed to give any colonies. Editing efficiency increased with arm length from 800 bp to 1 kb, with the 1 kb design achieving the highest recovery of correct deletions. These results guided the subsequent experiments, where 1 kb homology arms were used as the default design for Cas9-assisted gene deletion in *A. kunkeei* strains.

For each modified strain, plasmid curing was performed by growing in non-selective medium overnight. Plasmid curing was confirmed by colonies from highly diluted non-selective cultures and testing their growth on double-antibiotic plates, with non-growing colonies scored as plasmid-free. Plasmid-free strains were used for whole-genome sequencing or downstream phenotypic assays. Interestingly, curing was challenging when either pMER or pMECK was present, but when both plasmids were present, curing in the absence of selection yielded more than ~60% plasmid-free strains (Table S8). Overall, the initiator–effector design not only simplified vector construction but also facilitated easier curing.

### Gene replacement

To extend the application beyond gene deletions, we performed a knock-in gene replacement. As a demonstration, the complete coding region of *gntT* was replaced with the mCherry reporter gene (Fig. S6A). We used the pMECK plasmid backbone carrying a 1 kb cassette with a codon-optimized mCherry sequence flanked by the homology arms of the *gntT* gene (Fig. S6B). A transcriptional terminator was included to prevent read-through from neighboring genes. The sgRNA design placed the PAM on the non-coding strand (the sgRNA targeted the coding strand), which yielded efficient editing in our deletion experiments and was therefore adopted for the knock-in strategy. Following transformation into pMER-carrying A1401 cells and induction with sakacin peptide, colonies were recovered, and the plasmid was cured. The expected replacement was observed in 16 out of 16 screened colonies (~100% efficiency, Fig. S6C), consistent with the successful generation of Δ*gntT*::mCherry mutant. Whole-genome sequencing of representative clones confirmed precise replacement in the mutant strains, consistent with the corresponding fluorescence measurements (Fig. S6D and E). These results establish that the system can efficiently replace native genes with heterologous sequences in *A. kunkeei*.

### *In situ* gene modification using single-stranded oligonucleotides and double-stranded repair templates

**Fig 3:**
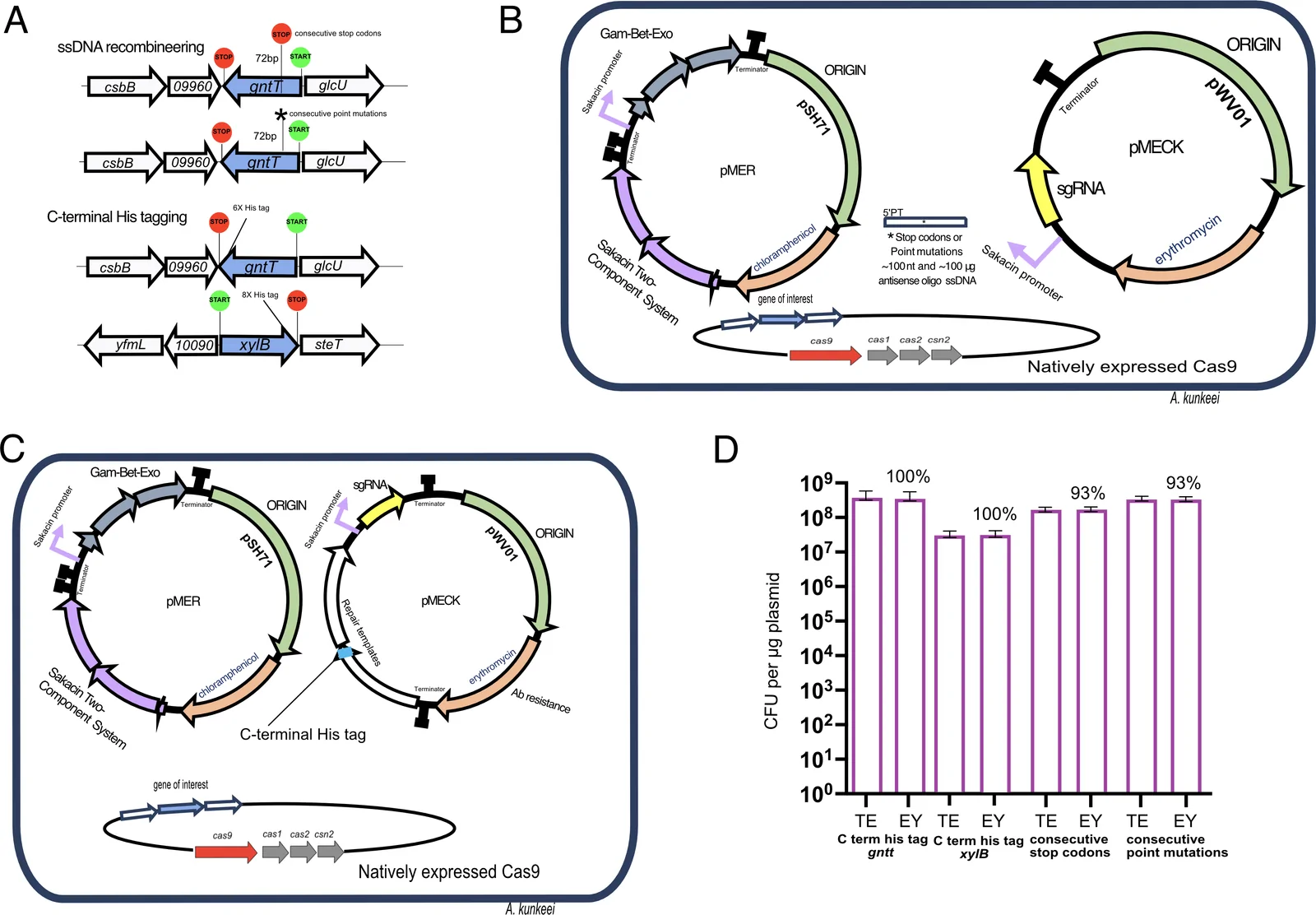
Dual-plasmid endogenous CRISPR-Cas genome editing for *in situ* modifications. (**A**) Schematic depicting the *in situ* modifications performed on *gntT* and *xylB* genes. The target gene is highlighted in blue, whereas the flanking genes are white. The start and stop codons are highlighted as colored circles. (**B and C**) The pMECK plasmid encodes the sgRNA under the control of a sakacin-inducible promoter, and either contains plasmid-borne double-stranded repair templates for His-tag insertion or is co-transformed with synthetic single-stranded oligonucleotides for recombineering. To prevent cleavage of the donor DNA during repair, PAM sequences were mutated within the repair template. For oligonucleotide-based editing, 5′-phosphorothioate-modified single-stranded DNA with short homology arms (45 bp on each arm, ~100 µg) was used. The pMER plasmid carries the *gam–bet–exo* recombination machinery and sakacin two-component kinase-regulator system to facilitate homology-directed repair and regulate cognate promoters, respectively. Both plasmids are maintained under compatible replication origins (pWV01 and pSH71) and selected with erythromycin or chloramphenicol, respectively. Upon induction with the sakacin signaling peptide, expression of the sgRNA and recombinase enables endogenous Cas9-mediated cleavage followed by precise repair. This system was used to introduce consecutive stop codons, point mutations in the *gntT* gene, C-terminal 6×His tagging of *gntT*, and 8×His tagging of *xylB*. Successful tagging was confirmed by whole-genome sequencing, while SNP introductions were validated by Sanger sequencing. (**D**) Editing efficiencies are presented as colony-forming counts per microgram pMECK plasmid and expressed as mean ± standard deviation, where TE represents the apparent transformation efficiency (or final viable colony yield), and EY denotes the editing yield. As editing experiments involve extended recovery and selective enrichment steps, the resulting transformation efficiencies appear higher than true transformation efficiencies. CFU was calculated as the number of colonies obtained per microgram of pMECK plasmid DNA transformed following all recovery, induction, and selection steps, whereas editing yield was determined as the percentage of edited colonies relative to the total number of transformants.

To enable precise genomic modifications, we applied the initiator–effector framework to perform consecutive point mutations, premature stop codon insertion, and C-terminal epitope tagging in *A. kunkeei* A1401 (Fig. 3A and D). Building on the *rpoB* recombineering results, we tested single-stranded oligonucleotide–mediated editing at the *gntT* locus. Cells carrying pMER were co-transformed with pMECK encoding an sgRNA targeting the coding strand near the desired edit site and a synthetic single-stranded oligonucleotide (~100 nt, 100 µg, 5′ phosphorothioate-modified) designed either to introduce premature stop codons or consecutive point mutations disrupting the ATAAA PAM located 72 bp downstream of the start codon (Fig. 3B). Both modifications were obtained with ~93% efficiency, and all verified edits were confirmed by Sanger sequencing (Fig. 3D; Fig. S7A). Notably, consecutive point mutations consistently yielded higher colony numbers than stop codon insertions, despite both disrupting *gntT* function.

For C-terminal tagging, *gntT* was modified using pMECK with repair templates encoding a 6×His tag immediately upstream of the stop codon (Fig. 3C). A single codon substitution within the PAM was introduced in the repair template to prevent Cas9 cleavage. sgRNAs targeting the coding strand near the stop codon yielded 100% tagging efficiency, whereas targeting the noncoding strand produced no colonies (Fig. 3D). Extending this approach, an 8×His tag was introduced at the C terminus of the cytosolic *xylB* gene using a coding-strand–targeting sgRNA (GTAAA PAM), resulting in ~100% tagging efficiency (Fig. 3D; Fig. S7B). The tagged protein was successfully purified by Ni-NTA chromatography and SDS-PAGE (Fig. S8). Whole-genome sequencing of plasmid-cured strains confirmed precise tag insertion. Together, these results demonstrate that the platform supports both efficient epitope tagging and precise nucleotide-level genome modifications in *A. kunkeei*.

### CRISPR interference in *A. kunkeei*

**Fig 4:**
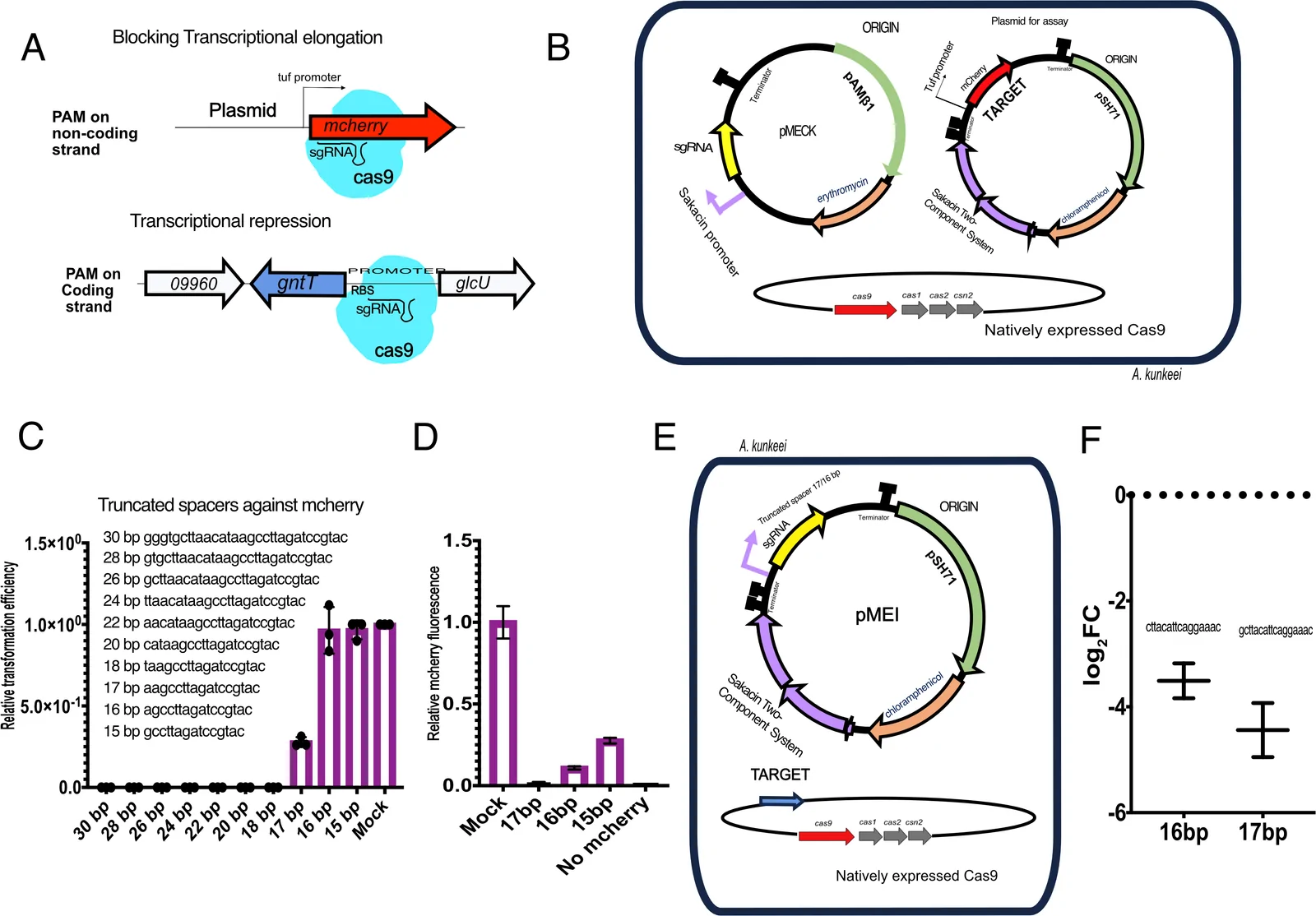
CRISPR interference. (**A**) Schematic depicting the CRISPRi in the form of blocking transcriptional elongation for the *mCherry* reporter cassette in a plasmid and transcriptional repression on chromosomal *gntT*. For the plasmid-encoded *mCherry* reporter, the protospacer and PAM were positioned on the non-coding strand, whereas for chromosomal *gntT* repression, the protospacer and PAM were positioned on the coding strand. (**B**) The pMECK plasmid encodes sgRNAs of varying spacer lengths under the control of a sakacin-inducible promoter to enable programmable repression of target genes. A second plasmid constitutively expresses the *mCherry* reporter from the tuf promoter. Upon induction with the sakacin signaling peptide, the sgRNA is expressed and guides the endogenous Cas9 to the *mCherry* locus, where it binds without cleaving the DNA, resulting in transcriptional interference. (**C**) Transformation efficiencies are presented as colony-forming counts per microgram pMECK plasmid expressed as mean ± standard deviation, normalized to the non-targeting control (Mock). A lower transformation efficiency indicates higher CRISPRi. (**D**) Relative *mCherry* fluorescence, normalized to wild-type *mCherry* levels, is shown for truncated spacers ranging from 17 to 15 nucleotides in length, demonstrating spacer length–dependent repression efficiency. (**E**) Self-contained pMECK derivative (pMEI) targeting the promoter region upstream of the ribosome binding site of *gntT*. (**F**) qRT-PCR analysis confirmed complete transcriptional repression using 17 bp and 16 bp spacers, reported as log_2_ fold change relative to Mock spacer, indicating effective CRISPRi-mediated gene silencing at the endogenous chromosomal locus.

We assessed whether the endogenous Cas9 in *A. kunkeei* A1401 could be adapted for transcriptional repression. For this purpose, we repurposed the natively expressed Cas9 nuclease with progressively truncated guide spacers (59) in sgRNA design, which abolishes DNA cleavage while retaining DNA-binding capacity (Fig. 4A). For plasmid-based CRISPRi, two plasmids were constructed: (i) a pSH71–chloramphenicol resistance marker vector carrying the two-component sakacin induction system and a constitutively expressed mCherry reporter was used as the target for repression and (ii) a compatible pAMβ1–erythromycin resistance marker pMECK plasmid which expresses sgRNAs from the sakacin promoter, with spacers of varying lengths (30–12 bp) targeting the mCherry coding sequence in the target plasmid (Fig. 4B). Despite the original spacer being 30 bp, Cas9-mediated cleavage still occurred with spacers as short as 18 bp (Fig. 4C). This cleavage activity was abolished at ≤17 bp as evidenced by the progressive increase in the number of colonies on the double-antibiotic plates. Consistent with these findings, fluorescence measurements of mCherry expression mirrored the transformation efficiency data, with the strongest repression at 17 bp, followed by 16 bp, and thereafter the effect subsided downward (Fig. 4D).

To extend this approach to chromosomal gene repression, we introduced a self-contained pMECK derivative (pMEI) into wild-type A1401 in which the two-component sakacin system and the *gntT*-targeting sgRNA cassette resided on the same plasmid (Fig. 4E). For promoter-proximal targeting, the protospacer and PAM were positioned on the coding strand upstream of the ribosome-binding site. Promoter-targeting CRISPRi is generally considered strand-independent (59). Transcript quantification done using quantitative RT-PCR showed that promoter-proximal targeting with 17 bp spacers achieved full repression, with 16 bp spacers showing a similar knockdown level (Fig. 4F; Table S9). Together, these results demonstrate that the *A. kunkeei* Cas9 can be repurposed for CRISPRi against both plasmid-borne and chromosomal targets, with repression efficiency influenced by spacer length, target location, and strand orientation.

## DISCUSSION

As a dominant honeybee foregut symbiont, *A. kunkeei* is a promising candidate for paratransgenic strategies aimed at bee health, microbiome modulation, or pathogen suppression (33–45). Harnessing endogenous systems offers a practical route to overcome genetic manipulation barriers in non-model bacteria (4, 32). By leveraging native PAM recognition and guiding RNA maturation pathways, such approaches promise improved host compatibility while reducing burden. However, *A. kunkeei* has remained inaccessible to functional genomics precisely because these strategies were lacking. Consistent with recent advances in lactic acid bacteria, where both heterologous and endogenous CRISPR-based editing strategies have been developed, our study extends endogenous CRISPR-Cas genome engineering to *A. kunkeei*. The Type II-A CRISPR-Cas system was identified in ~91% of strains, suggesting that similar approaches may be applicable across much of the *A. kunkeei* population.

Our work integrates two key features: exploitation of the endogenous Cas9 nuclease and an initiator–effector configuration that coordinates recombinase and guide RNA expression for inducible, targeted genome modification. The inducible design not only improved editing efficiency during the transformation but also provided practical benefits in plasmid construction since the sakacin system is known to be inactive in the *L. lactis* subcloning host (6). A functional type II-A CRISPR-Cas system was conserved in both gene arrangement and array structure across phylogroups, providing a strong genetic foundation for the development of endogenous genome-editing tools. Defining strain-specific PAM preferences and confirming precise crRNA/tracrRNA processing established the sequence rules and RNA architecture for target recognition. Plasmid-interference assays verified that constitutively expressed Cas9 exhibited stringent PAM specificity, with a five-nucleotide core limiting flexibility, yet confirming its suitability for potent *in vivo* targeting. The sakacin-inducible recombinase module supported efficient oligo-directed mutagenesis, demonstrating its effectiveness as an integrated component of the endogenous Cas9 editing system. Overall, our findings support that endogenous CRISPR-Cas systems can be adapted into effective editing platforms through a relatively simple workflow involving PAM characterization, guide design, and inducible recombineering.

The initiator–effector configuration comprised two rolling circle vectors: (i) the pMER plasmid carrying the sakacin-inducible recombinase module and (ii) the pMECK plasmid carrying the guide RNA cassette and repair template. This arrangement allowed coordinated expression of the recombinase and the sgRNA during editing, ensuring that Cas9-mediated cleavage occurred in the presence of active recombineering functions. This dual configuration proved effective across a wide spectrum of genetic modifications, from small nucleotide substitutions and epitope tagging to complete gene deletions, demonstrating efficiencies comparable to CRISPR-Cas9 systems reported in other lactic acid bacteria. The system produced scarless gene deletions and further precise modifications, with minimal off-target effects. Beyond small-gene knockouts and precise *in situ* modifications, the native *A. kunkeei* Cas9 demonstrated the single-sgRNA mediated deletion of ~25 kb, a scale at which most bacterial systems are reported to require either dual sgRNAs or Cas3-based nucleases. The efficiency observed here underscores the robustness of this enzyme for both compact and extended chromosomal edits. In addition to its endonuclease activity, spacer truncation (59) provided a straightforward way to repurpose the same system for CRISPRi, enabling targeted transcriptional repression without DNA cleavage. For chromosomal gene repression, a self-contained pMECK derivative (pMEI) can be used, in which the two-component sakacin system and the sgRNA cassette reside on the same plasmid. Repression could be modulated by adjusting spacer length, target position, and strand orientation, offering a practical route to study essential or conditionally essential genes where knockouts are not feasible. This expands the functional scope of the platform beyond genome editing to dynamic gene regulation within the same genetic framework.

Despite the high editing efficiencies achieved in this study, an important limitation of the system is the dependence on PAM availability. In contrast to commonly used Cas9 homologs such as SpyCas9 (25), which recognize short PAMs, the *A. kunkeei* Cas9 variants require extended 5–6 nt PAM sequences. Consequently, suitable target sites occur less frequently in the genome, which may restrict editing at certain loci and limit the introduction of precise nucleotide substitutions at arbitrary positions. In addition, the efficiency of isolated single-nucleotide substitutions remains to be systematically assessed, as editing outcomes may differ from those obtained with consecutive nucleotide substitutions. Future efforts to streamline the editing workflow and reduce dependence on heterologous genetic components may further expand the applicability of the system. In addition to establishing genome engineering in *A. kunkeei*, this study highlights several practical factors that influence successful genetic manipulation of non-model bacteria, including PAM selection, repair-template design, plasmid compatibility, and transformation efficiency. Collectively, these observations provide useful considerations for adapting endogenous CRISPR-Cas-based genetic tools in other experimentally challenging bacterial species. In summary, the development of a genetic system for *A. kunkeei* removes a long-standing technical obstacle in a key honeybee-associated bacterium and enables mechanistic interrogation of its roles in host health, microbial interactions, and environmental adaptation.

## MATERIALS AND METHODS

### Bioinformatic analyses

The genomes of 102 isolates of *A. kunkeei* were retrieved from NCBI (National Center for Biotechnology Information). Strain designations and accession numbers are described in Table S1. CRISPR arrays and associated Cas proteins were identified using CRISPRCasFinder (54) with default parameters. Repeat and spacer sequences were extracted from identified CRISPR arrays, and consensus repeats were summarized per phylogroup. The sequence conservation was visualized using WebLogo (60), ensuring coverage of several spacers. For each Cas protein, a multiple alignment was performed using MMseqs2 (61) in easy-search mode with a Smith-Waterman alignment-mode, and percent identity was calculated based on alignment coverage thresholds of ≥50% for both query and target sequences. For the Cas9 ortholog comparison, the coverage threshold was reduced to ≥15% to accommodate the greater sequence divergence among representative Cas9 proteins from different bacterial species. Protospacer-adjacent motifs were predicted with Spacer2PAM (62), which aligns spacers to phage and bacterial genomes to infer enriched flanking sequences (excluding eukaryotes). For Type II-A systems, the 10-nucleotide flanking regions on the 3′ ends of protospacer sequences were additionally aligned manually to confirm PAM enrichment. Candidate PAM motifs were subsequently searched genome-wide with the FIMO tool (63) from the MEME suite. Hits were classified as coding, noncoding, upstream (±200 bp), or intergenic according to coding sequences and features annotated in the corresponding GenBank files.

### Bacterial strains

*A. kunkeei* strains A1401, H3B2-02X, and H2B1-05J were used as the parent strains for the genetic engineering experiments based on growth rates, absence of intact phages (38), and transformation abilities. For cloning experiments, *Lactococcus lactis* NZ9000 was used. Antibiotics were added in the following concentrations: Erythromycin (20 µg/mL for *A. kunkeei* and 5 µg/mL for *L. lactis*) and chloramphenicol (6 µg/mL for *A. kunkeei* and 5 µg/mL for *L. lactis*). The strains were routinely cultivated in De Man–Rogosa–Sharpe (MRS) medium with 5 g/L fructose (f-MRS) and incubated statically at 35°C with 5% CO_2_.

### Construction of vectors for CRISPR-Cas9 editing in *A. kunkeei*

Plasmids pVPL3017 (rolling circle pSH71 origin) and pVPL3115 (rolling circle pWV01 origin) (5), kindly provided by the Jan-Peter van Pijkeren group (University of Wisconsin–Madison), and plasmid pTRKH2 (theta-replicating pAMβ1 origin, Addgene #71312), were used as template DNA to construct derivatives for use in *A. kunkeei*. Plasmid pSIP411 (53) was kindly provided as a gift from the Geir Mathiesen group. All plasmids and strains generated or used in this study are described in Table S10. All oligonucleotides and synthetic DNA fragments are listed in Table S11. Unless otherwise stated, all plasmid assemblies were performed by Gibson assembly using overlapping PCR amplicons.

The recombineering plasmid pMER was derived from the backbone of pVPL3017 (pSH71 origin, chloramphenicol resistance). The sakacin two-component regulatory system (pSIP, Histidine kinase, Response regulator), terminators, and PorfX promoter were amplified from the pVPL3017 backbone and assembled with the recombinase operon. The recombinase operon, consisting of the PLE3 phage–derived *bet*, *exo*, and *gam* genes, was obtained as a synthetic construct (Thermofisher) with flanking overlaps to the backbone.

The CRISPR plasmid pMECK (pWV01 origin, erythromycin resistance marker) origin was derived from pVPL3115 and the erythromycin resistance gene from pSIP411. The sgRNA cassette was placed under the control of the PorfX promoter. For gene deletion experiments, homology arms (200 bp–1 kb per arm) were PCR-amplified from genomic DNA and assembled with the sgRNA cassette. Variants of pMECK were also prepared to evaluate designs: (i) different repair templates based on the gene to be modified, (ii) the sgRNA cassette was driven by a constitutive promoter to compare against sakacin-inducible expression, (iii) mock sgRNA with repair templates, (iv) repair templates without any sgRNA, and (v) origin was changed to pAMβ1 and sgRNA with different truncated spacers tested for transcriptional repression experiments.

### Growth and cultivation of strains

For growth experiments, a loopful culture of the representative strains was collected from glycerol stocks stored in −80°C, was streaked on f-MRS agar, and incubated overnight at 35°C with 5% CO_2._ A single colony was chosen to inoculate 5–10 mL of f-MRS liquid broth and incubated overnight at 35°C, 5% CO_2_ statically. The final biomass or progress of growth of the batch culture was measured in terms of optical density (OD) at 600 nm wavelength. Precultures in f-MRS usually support an OD of 1.5–1.8. A fixed volume of preculture was chosen to inoculate the main culture, such that the starting OD is 0.1. A total 0f 1 mL of culture was harvested at regular intervals for OD measurement.

### Electroporation and screening

Transformations for the *A. kunkeei* strains were done as described previously with a few modifications (46, 64). Briefly, overnight cultures grown statically in f-MRS media at 35°C were used to inoculate 200 mL of f-MRS with 2% glycine to an initial OD_600_ > 0.1. Appropriate antibiotic concentrations were added to the pre-culture as well as to the main cultures based on the plasmids (pMER, pMECK, etc.) used. Cells were harvested at mid-exponential phase (OD_600_ = 0.5–0.6), chilled on ice for 10–15 min, and centrifuged in 50 mL Falcon tubes (4,000 rpm, 4°C). The pellets were washed once with 1 mM cold MgCl₂, resuspended in 1 mL ice-cold sterile water, and transferred into 2 mL tubes. Each was centrifuged (12,000 × *g*, 3 min) and washed three times with 1 mL ice-cold sterile water, followed by one wash with 1 mL ice-cold 30% PEG 8000. Pellets were resuspended in 0.5 mL 30% PEG 8000, pooled, and aliquoted into cryotubes (0.2 mL each) for storage at −80 °C. Before transformation, the electrocompetent cells were thawed on ice. The cells were pre-treated with lithium acetate (LiAc) and DTT such that 0.25 mL of LiAc and 50 µL of 100 mM DTT were added to 0.2 mL of cells and incubated on ice for 30 min. The cells were then centrifuged to remove the salts and washed once with 1 mL ice-cold 30% PEG 8000. Finally, cells were resuspended with 0.2 mL of ice-cold 30% PEG 8000 and electroporation was performed using 100 µL of cells. Electroporation was performed using BioRad Gene Pulser with the built-in *E. coli* program with the following parameters (2.5 kV, 5 ms time constant, 25 µF capacitance) and using a 2 mm cuvette. All electroporations were performed with at least 2 µg of plasmids or 100 µg of oligonucleotides. Once pulsed, 0.9 mL of recovery media was immediately added to the cuvette and collected into a 50 mL Falcon tube. The composition of the recovery media was identical to the one described previously. The culture was recovered overnight at 35°C with an additional 4 mL of recovery media, and a fixed 200 µL of cells was spread on suitable antibiotic plates. The individual colonies were picked and patched on the respective antibiotic plate of the same concentration. The patch plate was used for screening the colonies using PCR and was used for inoculation in 5 mL f-MRS overnight culture with respective antibiotics for subsequent glycerol stocks (~15% wt/vol final concentration) preparation.

PCR was performed using Phusion Hot Start Flex polymerase (NEB), and the colony PCR template for *L. lactis* was prepared by heating (98°C for 25 min) colonies in 30 µL nuclease-free water. The colony PCR template for *A. kunkeei* was prepared using GeneReleaser (BioVentures) as per the manufacturer’s instructions and diluted 1:20 in nuclease-free water with a fixed volume of the diluent used as template. PCRs were designed based on the primers used, with the addition of MgCl_2_ (2 mM at the final 1× reaction concentration) for *A. kunkeei*.

### Small RNA-sequencing

Cultures were grown to the mid-exponential phase, harvested by centrifugation, stored in Ambion RNALater, and later lysed via hand-held homogenizer in Trizol (Life Technologies, Carlsbad, CA). Unless otherwise specified, all cultures were harvested during the mid-exponential phase at OD_600_ = 0.5–0.6. In total, RNA was purified from the 10 lysates using the Ambion Ribopure kit followed by DNase digestion. Total RNA was submitted to BGI Genomics and checked for RNA quality. Once the quality criteria were met, small RNA libraries were prepared with the UMI small RNA library kit with size-selection of fragments around 17–200 nucleotides in length. The libraries were sequenced on the DNBSEQ platform with a read length of 50 nucleotides in a single-end and unstranded setup. The data were received de-multiplexed and with adapter sequences, and low-quality reads were removed as well as filtered to exclude reads <15 nucleotides. The data were used for alignment to the reference genome using breseq (65) and visualized using Tablet genome viewer (66). Coverage plots depicting the crRNA and tracrRNA expression were generated with the custom scripts in Python.

Based on the small RNA-Seq data that confirmed boundaries for the tracrRNA and crRNAs, we designed a synthetic single-guide RNA cassette. The sgRNA, along with the sakacin PorfX or constitutive *tuf* promoter, appropriate spacer, and terminator, was synthesized as a DNA string project (Thermofisher) (Table S11). The sgRNA scaffold was designed based on the predicted crRNA duplex. The repeat-derived crRNA segment was retained up to four nucleotides adjacent to the bulge region, while the tracrRNA scaffold was retained beginning with the complementary four nucleotides and extending through the upper stem, nexus, and terminal hairpin. The crRNA and tracrRNA components were fused using a GAAA tetraloop, and seven thymidine residues (transcription termination signal) were appended downstream of the tracrRNA sequence (Table S11).

### Plasmid interference assay

Plasmid interference assays were performed to assess the activity of the natively expressed Cas9 and to determine PAM sequence requirements. Electrocompetent cells of representative *A. kunkeei* strains (A1401, H3B2-02X, and H2B1-05J) were prepared as described above. Test plasmids were constructed with the pSH71 origin of replication and a chloramphenicol resistance marker, carrying (i) a CRISPR array protospacer sequence with a cognate PAM, (ii) the protospacer sequence without PAM, or (iii) an empty vector control without protospacer and PAM. For cloning of protospacers, complementary oligonucleotides encoding the 30-bp target sequence along with terminators were annealed and inserted into the vector only backbone by Gibson assembly (NEB). Cells were electroporated with ~2 µg plasmid DNA and recovered in f-MRS broth at 35°C for 16 h. Transformation efficiency was determined by colony counts on selective plates (CFU). For PAM mutagenesis assays, single-nucleotide substitutions were introduced at positions 1–6 of the PAM, and transformation frequencies were compared to the wild-type PAM construct.

### Plasmid isolation and verification

Plasmids were propagated in *L. lactis* NZ9000 as the intermediate cloning host. When *E. coli* was used, plasmid preparations frequently yielded mixed populations within the same colony, consisting of both intact plasmids and truncated derivatives. For plasmid extraction, overnight cultures were grown in M17 broth supplemented with 0.5% glucose and the appropriate antibiotic. Plasmid DNA was isolated using the PureYield Plasmid Miniprep System (Promega) according to the manufacturer’s instructions. Prior to extraction, overnight cultures were washed with THMS buffer (Tris-HCl, MgCl₂, sucrose) and treated with lysozyme (50 mg/mL for ~30 min) (5). For *A. kunkeei*, the same protocol was followed with a starting volume of 40 mL per column, compared to 20 mL for *L. lactis*. DNA concentrations were determined with a Nanodrop spectrophotometer and verified by agarose gel electrophoresis. Whole-plasmid sequencing was performed by Plasmidsaurus using Oxford Nanopore Technology, followed by custom analysis and annotation to confirm sgRNA cassettes, recombinase operons, and homology arm junctions.

### CRISPR-Cas9 mediated gene knockout

Targeted gene deletions/modifications were carried out using a dual-plasmid configuration. Electrocompetent *A. kunkeei* cells were first transformed with pMER (pSH71 origin, chloramphenicol resistance), which carries the sakacin inducible *bet, exo*, and *gam* recombineering operon. After electroporation, cells were recovered in prewarmed recovery medium without antibiotics at 35°C statically for 12 h, and 200 µL was plated on f-MRS agar containing chloramphenicol. A single colony was restreaked, miniprepped, and the presence of the intact pMER plasmid was verified by plasmid isolation and sequence confirmation. Electrocompetent cells were then prepared from this verified pMER strain with induction as described above. For genome editing, pMECK (pWV01 origin, erythromycin resistance marker) carrying the target-specific sgRNA under sakacin control and repair templates with the desired flanking homology arms was introduced into the pMER host by electroporation. For ssDNA-based editing, pMECK carrying the target-specific sgRNA but no repair template was co-transformed with 100 µg of a synthetic single-stranded oligonucleotide (with phosphorothioate modifications at the 5′ end) containing the desired mutation. Following pulsing, cells were recovered in recovery medium without antibiotics at 35°C statically. After overnight recovery, the culture was diluted 1:10 into fresh f-MRS supplemented with chloramphenicol, erythromycin, and the sakacin peptide (10 ng/mL, MAGNSSNFIHKIKQIFTHR, 1 mg/mL synthesized from Sigma) and grown for an additional 16–24 h to ensure sustained induction of the sgRNA and recombinase. Cultures were then diluted appropriately and plated on f-MRS agar containing both antibiotics. Individual colonies were patched to a fresh double antibiotic plate and used as templates for screening. Plates were incubated at 35°C for 2–3 days for the colonies to develop. For CRISPR-Cas9 editing experiments, CFU counts represent surviving edited colonies after overnight recovery, induction, and double-antibiotic selection rather than the initial electroporation efficiency. Thus, these values are the final viable colony yield and are not directly comparable to baseline transformation efficiencies. For each assay, 16 colonies were selected at random and genotyped by colony PCR using primers 200 bp upstream or downstream of the homologous arms flanking the gene of interest.

### Plasmid curing

Once verification by PCR or WGS is done, a loopful of culture from the patch plate with either single or double antibiotic plates was inoculated in 5 mL f-MRS media without antibiotics for at least 18 h. A total of 200 µL of 1:100,000 diluted culture was spread onto f-MRS plates with no antibiotic. The single colonies were spotted onto f-MRS agar plates with and without antibiotics. Colonies that grew on plates without antibiotic but failed to grow on those with either single or double antibiotics were considered to be cured, following which one cured colony was sent for whole-genome sequencing. The genome sequences of edited strains were verified using hybrid Oxford Nanopore/Illumina sequencing performed by Plasmidsaurus. Reads from modified strains were analyzed with Breseq using default parameters, enabling confirmation of intended edits and detection of potential off-target mutations.

### CRISPR interference (CRISPRi)

CRISPRi was implemented by exploiting the endogenous Cas9 nuclease with truncated sgRNAs, a strategy that abolishes cleavage but retains DNA-binding capacity (59). For plasmid-based repression assays, a dual-plasmid system was used where wild-type *A. kunkeei* A1401 was transformed with the pSH71–chloramphenicol resistance marker vector carrying the sakacin two-component induction module and a constitutively expressed *mCherry* reporter. Verified clones were subsequently transformed with a pAMβ1–erythromycin plasmid encoding sgRNAs under sakacin control. Following overnight recovery, cultures were diluted 1:10 into fresh f-MRS medium containing chloramphenicol, erythromycin, and sakacin peptide (10 ng/mL), and incubated for an additional 16–24 h. Spacer sequences ranging from 30 to 12 bp were tested for their ability to modulate *mCherry* expression. After appropriate dilutions, cells were plated on double-antibiotic f-MRS, colony-forming units were recorded, and colonies were patched and inoculated for subsequent fluorescence measurements.

For chromosomal repression, a derivative of pMECK (pMEI) was constructed in which the sakacin two-component system and the sgRNA cassette were combined on the same plasmid. Wild-type *A. kunkeei* A1401 was transformed with pMEI constructs encoding promoter-proximal sgRNAs with truncated spacers (17, 16 bp). Induction was carried out with sakacin peptide (10 ng/mL), and repression was quantified by qRT-PCR of target gene transcripts.

### qRT-PCR

qRT-PCR was performed with the Applied Biosystems StepOnePlus real-time PCR machine using the PowerUp SYBR Green PCR Master Mix to confirm the repression of the promoter. *frr* was used as an internal control to normalize the qRTPCR data. DNAse-treated total RNA to cDNA conversion was performed using Superscript IV Reverse Transcriptase (Thermofisher) following the manufacturer’s instructions. All experiments were performed in biological duplicates and technical triplicates (*n* = 6). The 2^−ΔΔCt^ method described previously was used to quantify expression fold changes (67).

### Genomic DNA extraction

The genomic DNA extraction of the *A. kunkeei* strain was performed using Qiagen DNeasy Blood and Tissue kit with minor modifications. Briefly, 1.5 mL overnight culture (~1.5 OD) was harvested by centrifugation at 8,000 rpm for 2 min. The supernatant was discarded, and the pellet was resuspended in 180 µL enzymatic lysis buffer (preadded with 20 mg/mL lysozyme to aid in lysing the cell walls). The resuspension was incubated at 37°C for 45 min. The subsequent steps are followed as mentioned in the protocol. The final step involved elution using preheated nuclease-free water at 56°C. The samples were checked for quality using Nanodrop, concentration measured using Qubit, and sent to the Plasmidsaurus facility for whole-genome sequencing with either nanopore or hybrid option. If detection PCRs failed, we performed genome sequencing of random colonies having both the plasmids as well as the cured ones.

### Fluorescent measurements

The flow cytometry analysis protocol was performed considering certain aspects from a previous study (68). Briefly, replicate colonies were inoculated with *mCherry* in the plasmid in 5 mL f-MRS media with suitable antibiotics and induction peptide. The following day, a fresh culture (5 mL f-MRS) was inoculated using a 1:100 dilution of the overnight culture along with suitable antibiotics and induction peptide and allowed to grow till an OD_600_ of 0.5. The cells were collected by centrifugation and washed twice with phosphate-buffered saline (PBS, pH 7.4) and resuspended in PBS. A 1:100 dilution of the PBS resuspended cells gave a good response. Flow cytometry was performed using a MACSQuant VYB (Miltenyi Biotec), with PBS as the sheath fluid. mCherry fluorescence was excited at 561 nm and detected using the dsRed dTomato channel. The median intensity values were utilized to determine the shift in fluorescence for each of the samples.

### *gusA* assay

Strains carrying the *gusA* reporter plasmid pSIP411 were streaked onto erythromycin plates, and single colonies were used to inoculate 5 mL f-MRS broth. Cultures were induced overnight with sakacin peptide at final concentrations ranging from 0 to 5 ng/mL. All assays were performed in biological triplicate. The following day, cultures were harvested at mid-log phase, OD_600_ was recorded, and 1 mL of cells was pelleted and washed once with 100 mM sodium phosphate buffer (40 mM NaH_2_PO_4_, 60 mM Na2HPO4). Pellets were resuspended in 750 µL GUS buffer (per 50 mL: 25 mL sodium phosphate buffer, 15 mL autoclaved water, 175 µL β-mercaptoethanol, 100 mg lysozyme, 5 mL 0.1 M KCl, and 5 mL 10 mM MgSO4) and incubated at 37°C for 30 min. Following lysis, 8 µL of 10% Triton X-100 was added, vortexed, and incubated on ice for 5 min. Enzymatic reactions were initiated by adding 80 µL of 4-nitrophenyl-β-D-glucuronide (4-NPG; 10 mg/mL in 50 mM sodium phosphate buffer) and incubated at 37°C for 10–30 min until a visible yellow color developed. Reactions were stopped by adding 300 µL 1M Na2CO3, and samples were centrifuged at maximum speed for 6 min. The samples were diluted 1:10 or 1:50 using autoclaved water, and absorbance was measured at 405 nm and 550 nm in a plate reader (Agilent Biotek Synergy H1), and *gusA* activity was calculated in Miller units using the formula:


$$Miller\;units=1,000\times \;\frac{OD_{405}-\left(1.75\times \;OD_{550}\right)}{t\times V\times \;OD_{600}}$$

where *t* is the reaction time (min) and *V* is the culture volume used (mL).

### Ni-NTA affinity purification of His-tagged XylB

One liter of A1401 cells expressing the His-tagged construct was harvested (OD_600_ = 0.5) by centrifugation and stored at −20°C overnight. The pellet was suspended in 20 mL His binding buffer (50 mM Tris-HCl, 500 mM NaCl, 0.1 mM EDTA, 5% glycerol, and pH 8.0) supplemented with lysozyme (100 mg/mL), DNAseA, EDTA-free protease inhibitor, and 5 mM β (2-) mercaptoethanol, and incubated at room temperature for 1 h. Cells were lysed in a flow cell disruptor (Constant Systems Ltd., Daventry, UK) at 40 psi for three cycles, and the lysate was centrifuged at 15,000 × *g* for 45 min at 4°C. The supernatant was mixed with 0.5 mL pre-equilibrated Ni-NTA agarose (Protino) in a gravity column and incubated for 30 min to allow binding of the His-tagged protein. The column was washed twice with 20 mL His wash buffer (50 mM Tris-HCl, 500 mM NaCl, 0.1 mM EDTA, 5% glycerol, 10 mM imidazole, and pH 8.0). The target protein was eluted in His elution buffer (50 mM Tris-HCl, 500 mM NaCl, 0.1 mM EDTA, 5% glycerol, 500 mM imidazole, and pH 8.0) and collected in 0.5 mL fractions. Protein concentration in each eluted fraction was measured using a Nanodrop spectrophotometer, and purity was assessed by SDS-PAGE.

### Statistics

All the data in the figures are expressed as mean ± standard deviation. Unless otherwise specified, all the experiments were conducted independently with replicates and under identical conditions.

## Data Availability

The transcriptomic data have been deposited in ArrayExpress and are accessible through the accession number E-MTAB-16234. The plasmid sequences of the backbone plasmids pVPL3017, pVLP3115, and pSIP411, the PAM hits, the spacer sequences, and images for Weblogo-based PAM consensus, the *gntT* and *rpoB* gene sequences, and the genome sequences of the engineered strains are available in the SciLifeLab Data Repository at https://doi.org/10.17044/scilifelab.30579986.

## SUPPLEMENTAL MATERIAL

Supplemental figures aem.00728-26-s0001.pdf Fig. S1 to S8.

Supplemental tables aem.00728-26-s0002.xlsx Tables S1 to S11.

ASM does not own the copyrights to Supplemental Material that may be linked to, or accessed through, an article. The authors have granted ASM a non-exclusive, world-wide license to publish the Supplemental Material files. Please contact the corresponding author directly for reuse.
