# Supplementary material for: Harnessing endogenous CRISPR-Cas9 for inducible genetic engineering of Apilactobacillus kunkeei
Source: Appl Environ Microbiol. 2026 Jul 13;92(8):e00728-26. doi: 10.1128/aem.00728-26 (PMC13488297; doi:10.1128/aem.00728-26)
Supplement: Supplementary file 1 [file aem.00728-26-s0001.pdf]

Supplemental Material

Supplemental Figures

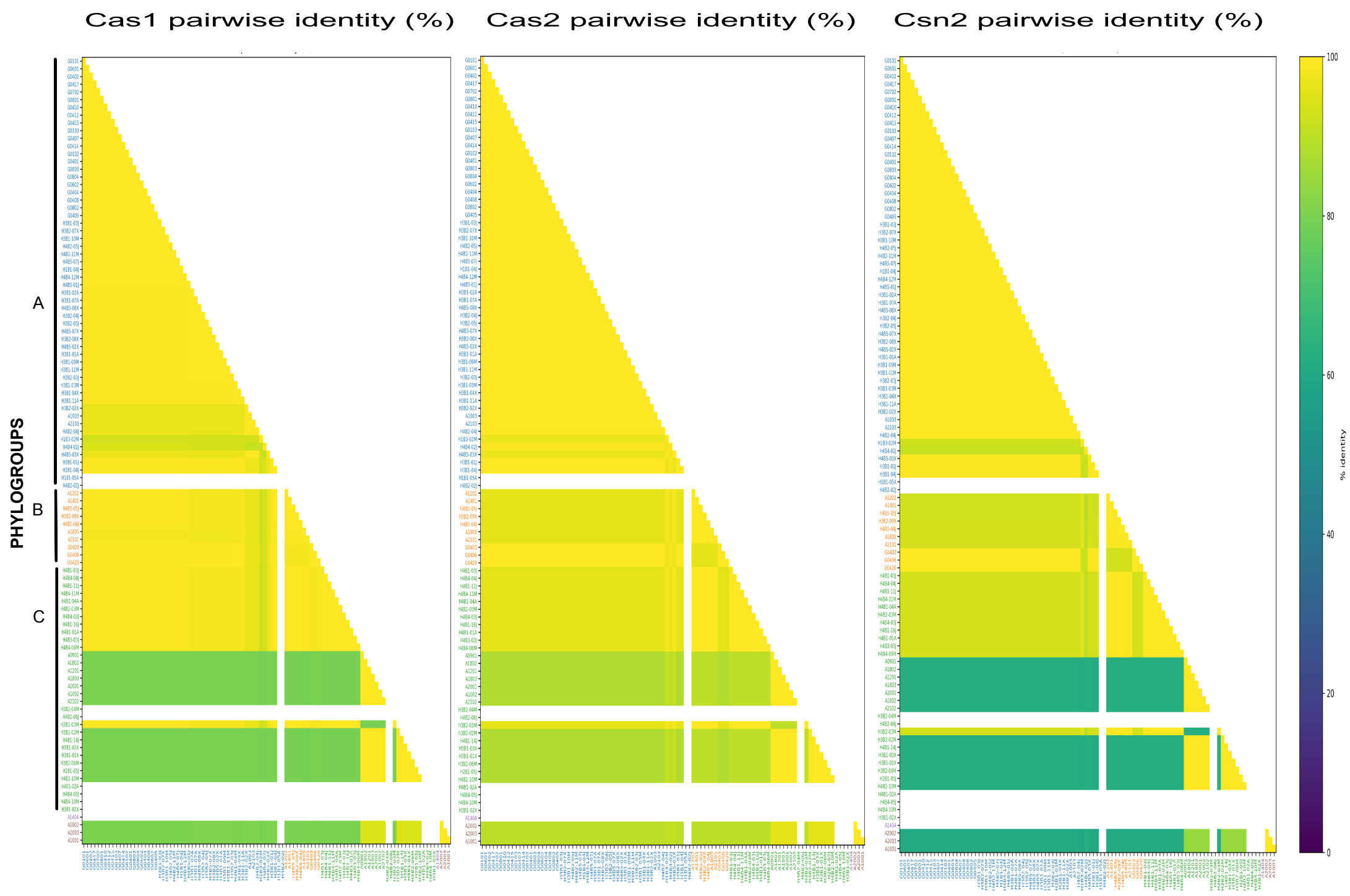

Fig S1: Pairwise protein sequence identity. The heatmap shows the lower triangular matrix of pairwise protein sequence identity for Cas1, Cas2 and Csn2 proteins across 102 *A. kunkeei* strains, plotted according to a predefined phylogroup order (A–F). Strain labels are colored by phylogroup classification: blue (group A), orange (group B), green (group C), purple (group E), and brown (group F). White spaces indicate either absence of a full-length protein or alignment coverage below 50%. The diagonal represents within-strain identity (100%).

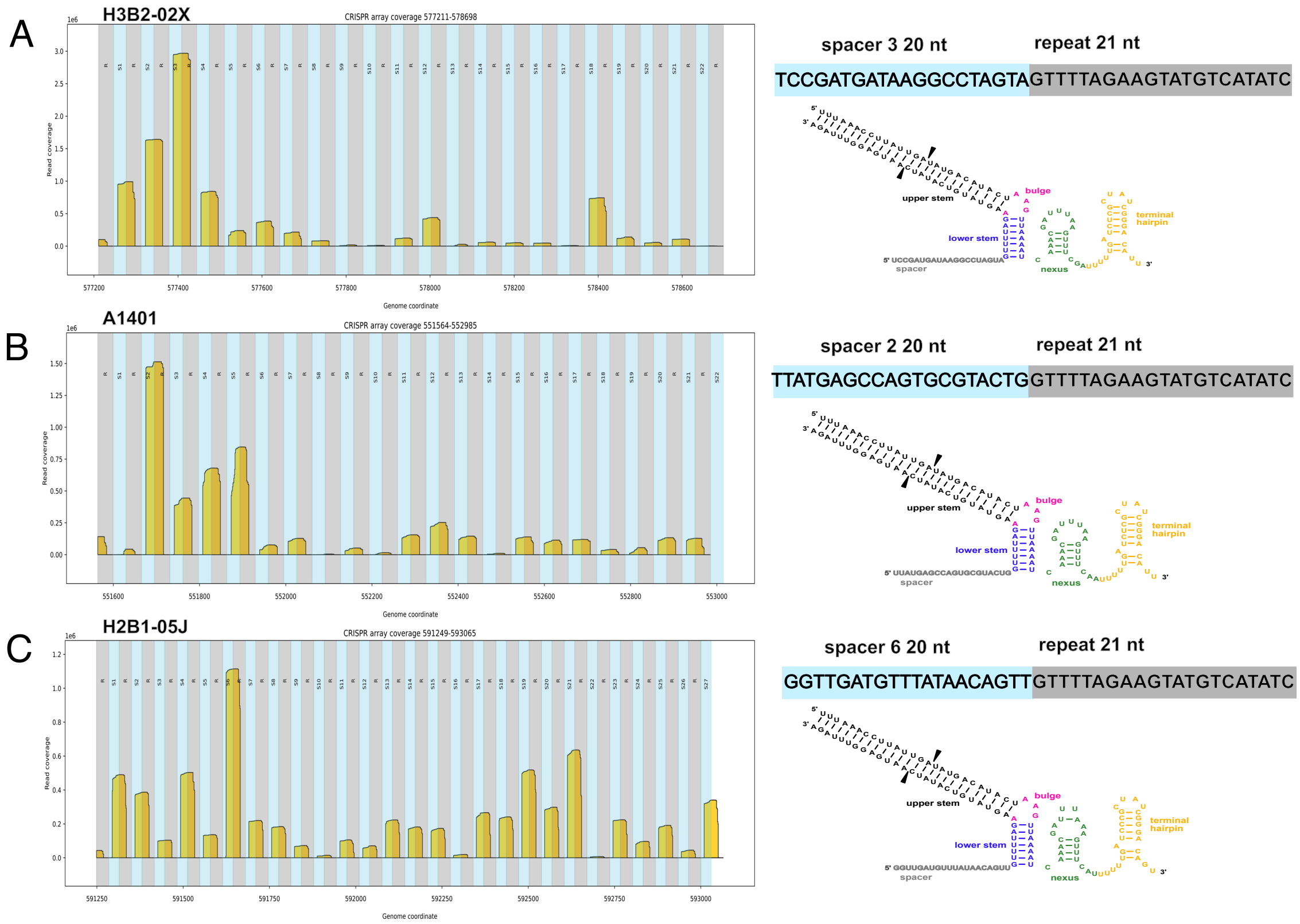

Fig S2: sRNA sequencing, boundary mapping and structure prediction. RNA-seq coverage plots showing transcriptional activity across the CRISPR array in strains A) H3B2-02X, B) A1401, and C) H2B1-05J. Read coverage is shown along the CRISPR array axis, with alternating spacer (S, light blue) and repeat (R, grey) units annotated. Predicted secondary structures of processed crRNAs corresponding to individual spacers derived from the CRISPR array. Each processed crRNA consists of a 5′ spacer-derived guide region followed by a 3′ repeat-derived stem-loop structure. Key structural features of crRNA:tracrRNA duplex, including the lower and upper stems, bulge, nexus, and terminal hairpin, are annotated based on conserved architecture characteristic of Type II-A CRISPR-Cas systems. Black arrow heads correspond to predicted RNAseIII cleavage site based on boundary mapping.

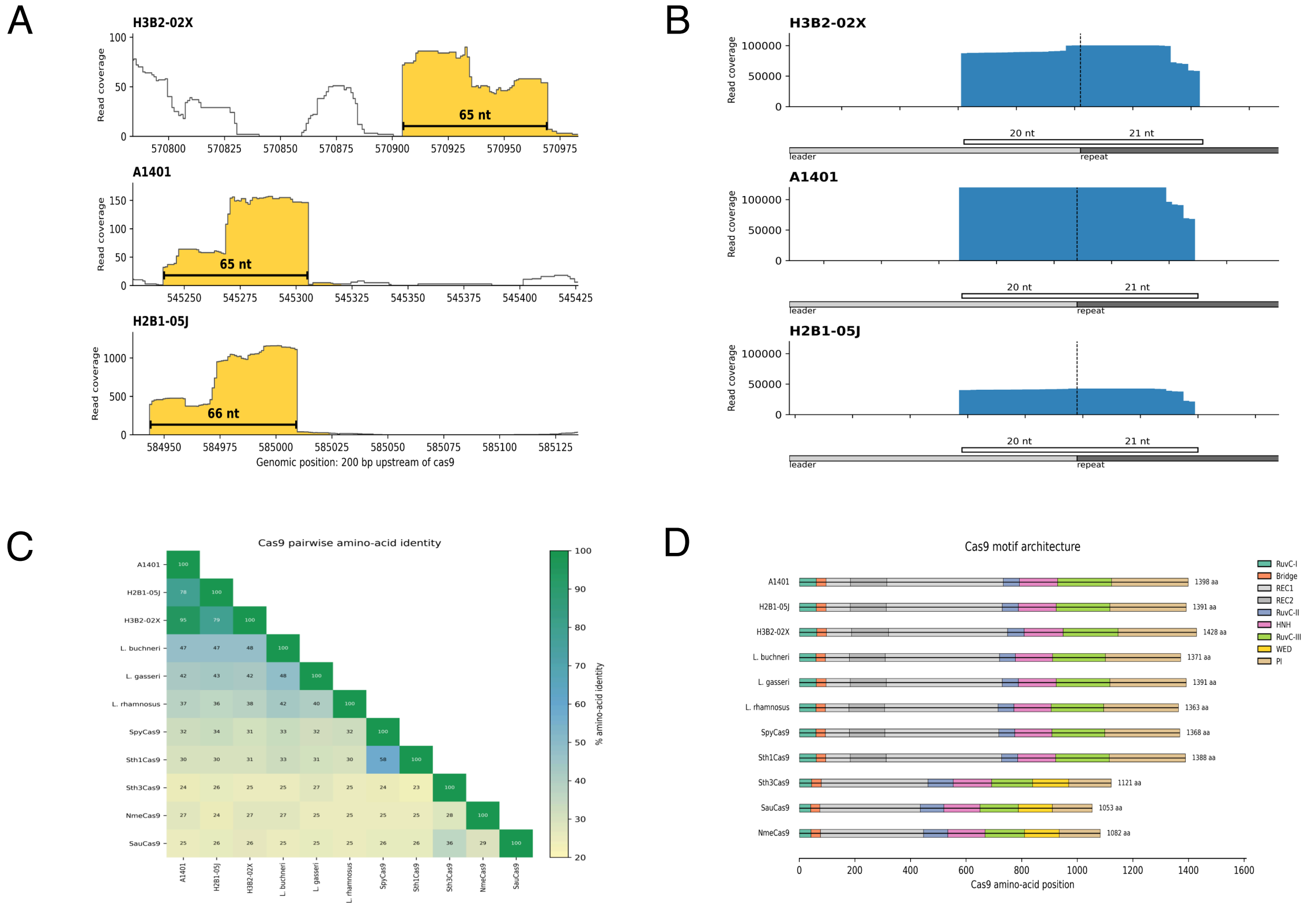

Fig S3: Comparative analyses. A) tracrRNA transcriptional activity across *A. kunkeei* strains. small RNA-seq read coverage plots showing full-length tracrRNA expression in strains A1401, H2B1-05J, and H3B2-02X, indicating active transcription of the CRISPR-Cas regulatory RNA. Yellow shading indicates the transcribed tracrRNA region. B) Small RNA-seq coverage across the leader–repeat junction of the CRISPR array in representative *A. kunkeii* strains. Coverage profiles support expression of the CRISPR-associated 5' end of leader RNA (ldrRNA). The dashed line indicates the boundary between the 20-nt leader-derived region and the 21-nt repeat-derived region. C) Pairwise amino-acid identity matrix of *A. kunkeii* Cas9 proteins and representative Cas9 orthologs from lactic acid bacteria together with canonical SpyCas9, SauCas9 and NmeCas9 proteins. D) Comparison of Cas9 domain architectures. Conserved RuvC, REC, Bridge, HNH, WED and PAM-interacting (PI) domains were identified by sequence mapping against the experimentally characterized SpyCas9 (*Streptococcus pyogenes*) and SauCas9 (*Staphylococcus aureus*) reference proteins.

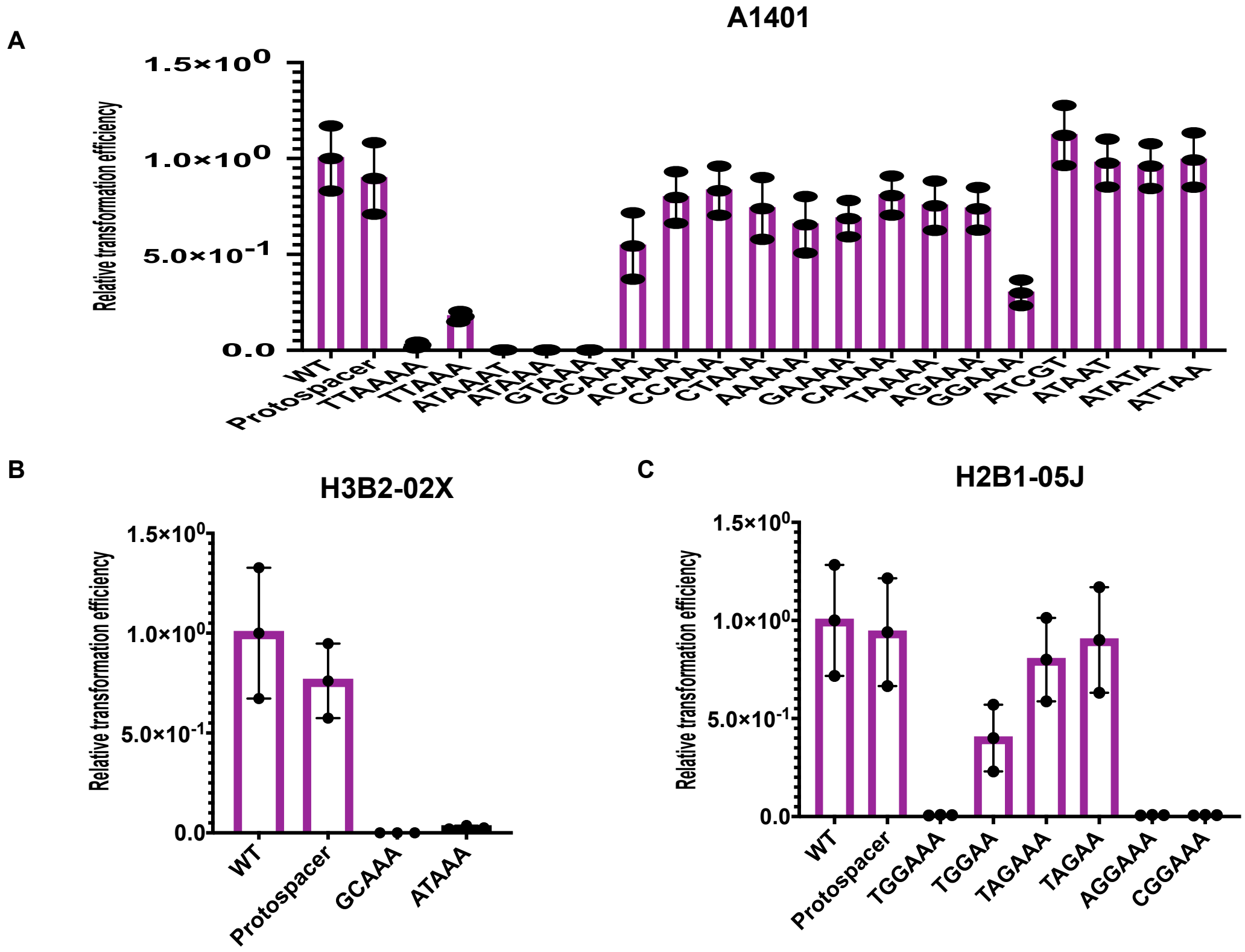

Fig S4: PAM specificity of the endogenous Cas9 in *A. kunkeei* strains. Relative transformation efficiencies measured using interference plasmids carrying single-nucleotide substitutions in the protospacer-adjacent motif (PAM) region for strains A) A1401, B) H3B2-02X and C) H2B1-05J. CFU was calculated as the number of colonies obtained per microgram of interference plasmids. Each bar represents the mean (± SD) of three biological replicates, normalized to the non-targeting wild-type control (WT).

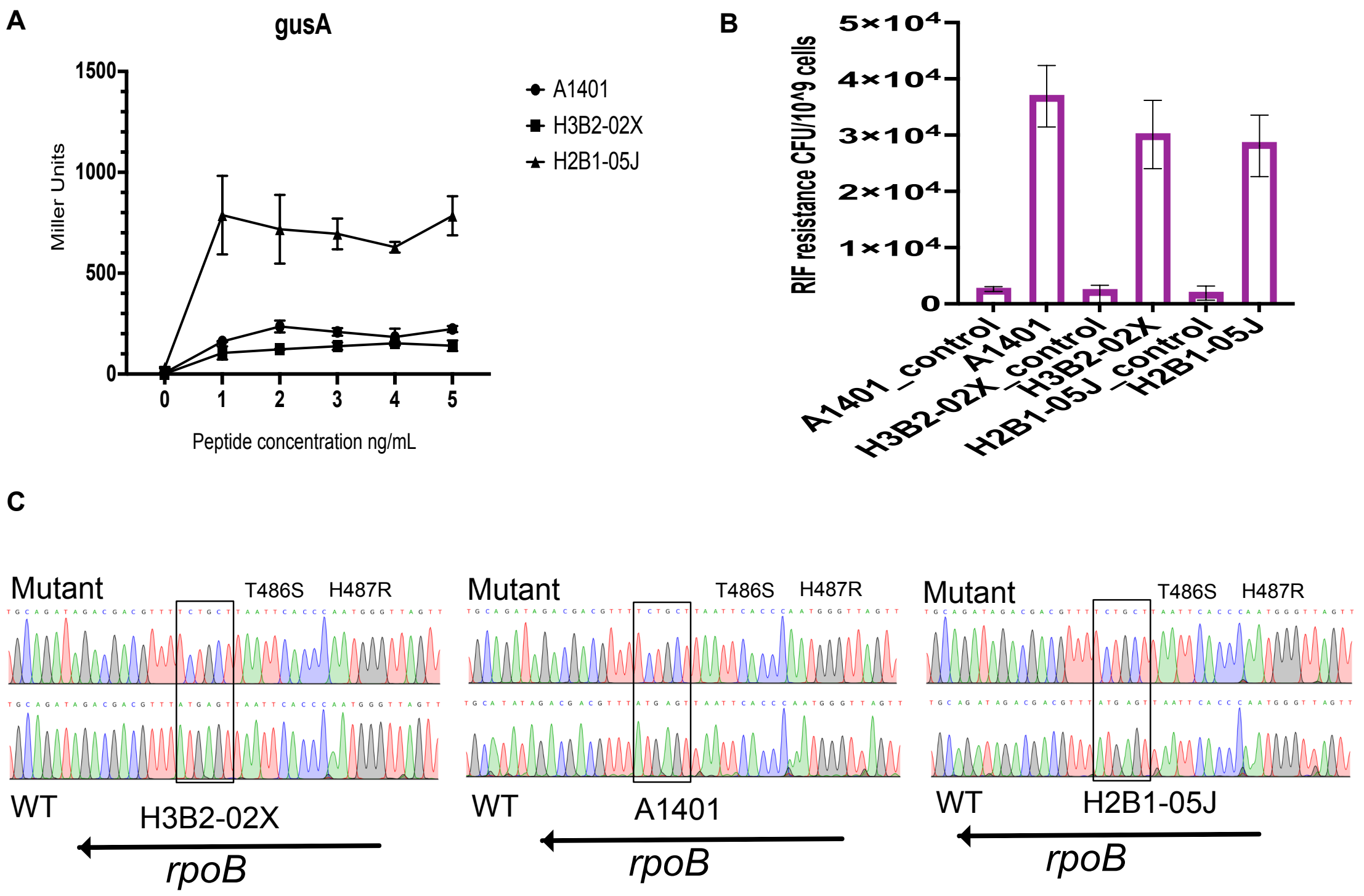

Fig S5: Sakacin inducible system. A) Miller assay showing dose-dependent activation of the sakacin-inducible promoter with *gusA* reporter gene for strains H2B1-05J, A1401 and H3B2-02X. B) Editing efficiency was quantified by introducing point mutations into the *rpoB* gene via phosphorothioate-modified single-stranded oligonucleotides, resulting in rifampicin-resistant colonies. Data are presented as rifampicin-resistant CFU per 10^9 cells, representing the mean of three independent experiments, with error bars indicating standard deviation. The frequency of rifampicin-resistant mutants reflects the efficiency of oligonucleotide-mediated genome editing. C) Chromatogram confirming *rpoB* point mutations (T486S and H487R) found in each strain relative to *rpoB* gene open reading frame (WT).

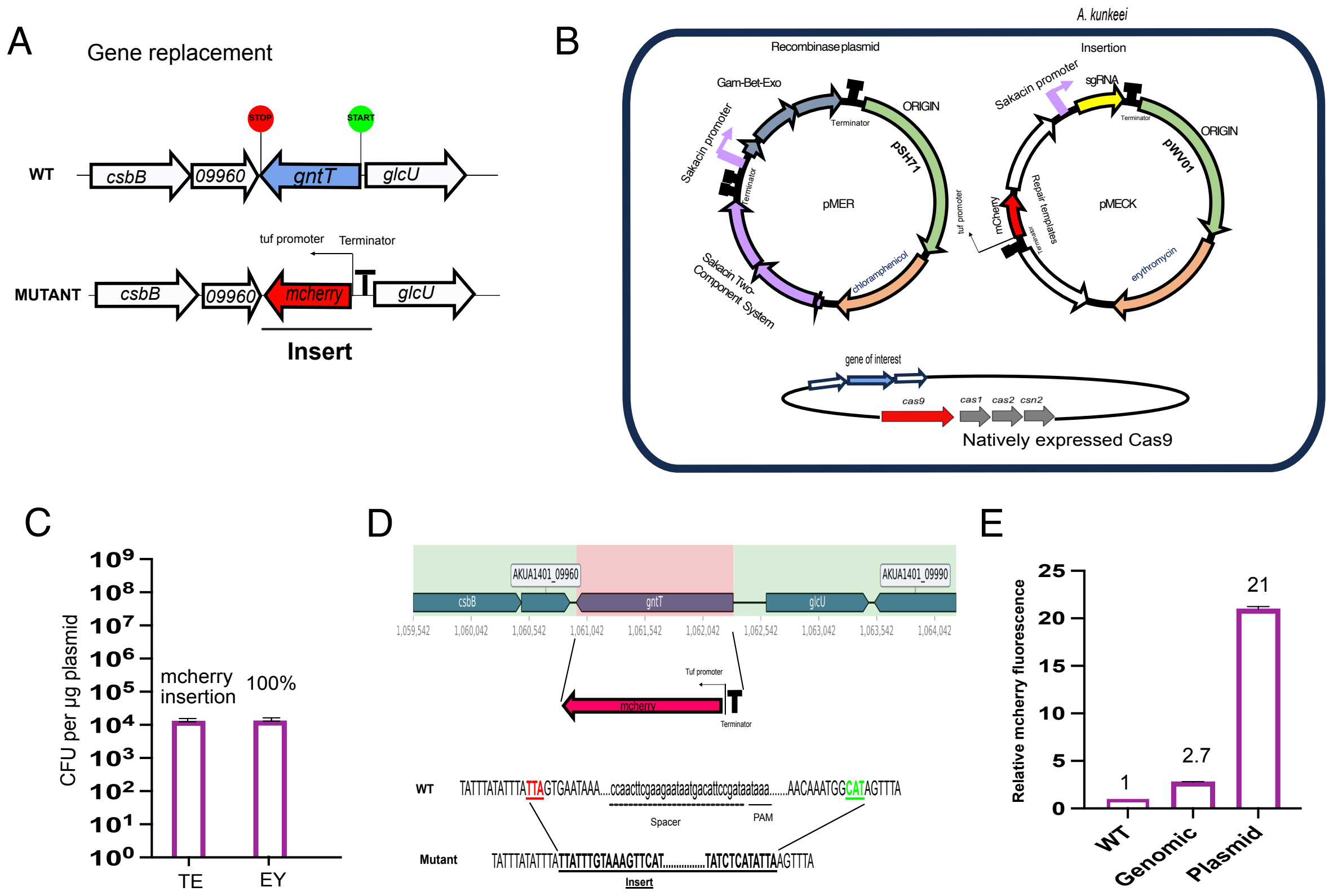

Fig S6: Dual-plasmid endogenous CRISPR-Cas genome editing for gene replacement. A) Schematic depicting the gene replacement of gene (*gntT* 1356 bp) with a terminator plus constitutive (*tuf*) promoter driven, codon optimized *mCherry* reporter cassette (referred as Insert). The target gene is highlighted in blue whereas the flanking genes are white. The start and stop codon are highlighted as colored circles. B) The pMECK plasmid contains the repair templates and encodes the sgRNA under the control of a sakacin-inducible promoter, enabling programmable targeting of chromosomal loci. The pMER plasmid carries the *gam–bet–exo* recombination machinery and sakacin two component kinase-regulator system to facilitate homology-directed repair and regulate cognate promoters, respectively. Repair templates are 315 bp upstream of the PAM for the *gntT* gene. Both plasmids are maintained under compatible replication origins (pWV01 and pSH71) and selectable with erythromycin or chloramphenicol, respectively. Upon induction with the sakacin signaling peptide, the sgRNA and recombinase genes are co-expressed, resulting in Cas9-mediated double-stranded DNA cleavage at the target locus. The system operates using the endogenous Cas9 machinery that cleaves and counter-selects WT cells and repair via homology-directed recombination which enabled complete replacement of *gntT* gene with codon-optimized constitutive promoter–*mCherry* reporter cassette. C) Editing efficiencies are presented as colony forming counts per microgram pMECK plasmid and expressed as mean ± standard deviation, where TE represents the apparent transformation efficiency (or final viable colony yield) and EY denotes the editing yield. As editing experiments involve extended recovery and selective enrichment steps, the resulting transformation efficiencies appear higher than true transformation efficiencies. CFU was calculated as the number of colonies obtained per microgram of pMECK plasmid DNA transformed following all recovery, induction and selection steps, whereas editing yield was determined as the percentage of edited colonies relative to the total number of transformants. D) DNA Feature Viewer visualization of sequenced genomes confirms targeted insertion at the *gntT* locus which are highlighted in pink. The nucleotides around the modification site are presented as text. E) Quantification of mCherry fluorescence following gene replacement. Relative fluorescence was measured in the wild-type strain (WT), the strain carrying a chromosomal *mCherry* insertion at the target locus (Genomic), and a strain expressing *mCherry* from a plasmid (Plasmid). Fluorescence was normalized to the wild-type control, demonstrating successful reporter expression from the chromosomal insertion, albeit at lower levels than plasmid-based expression.

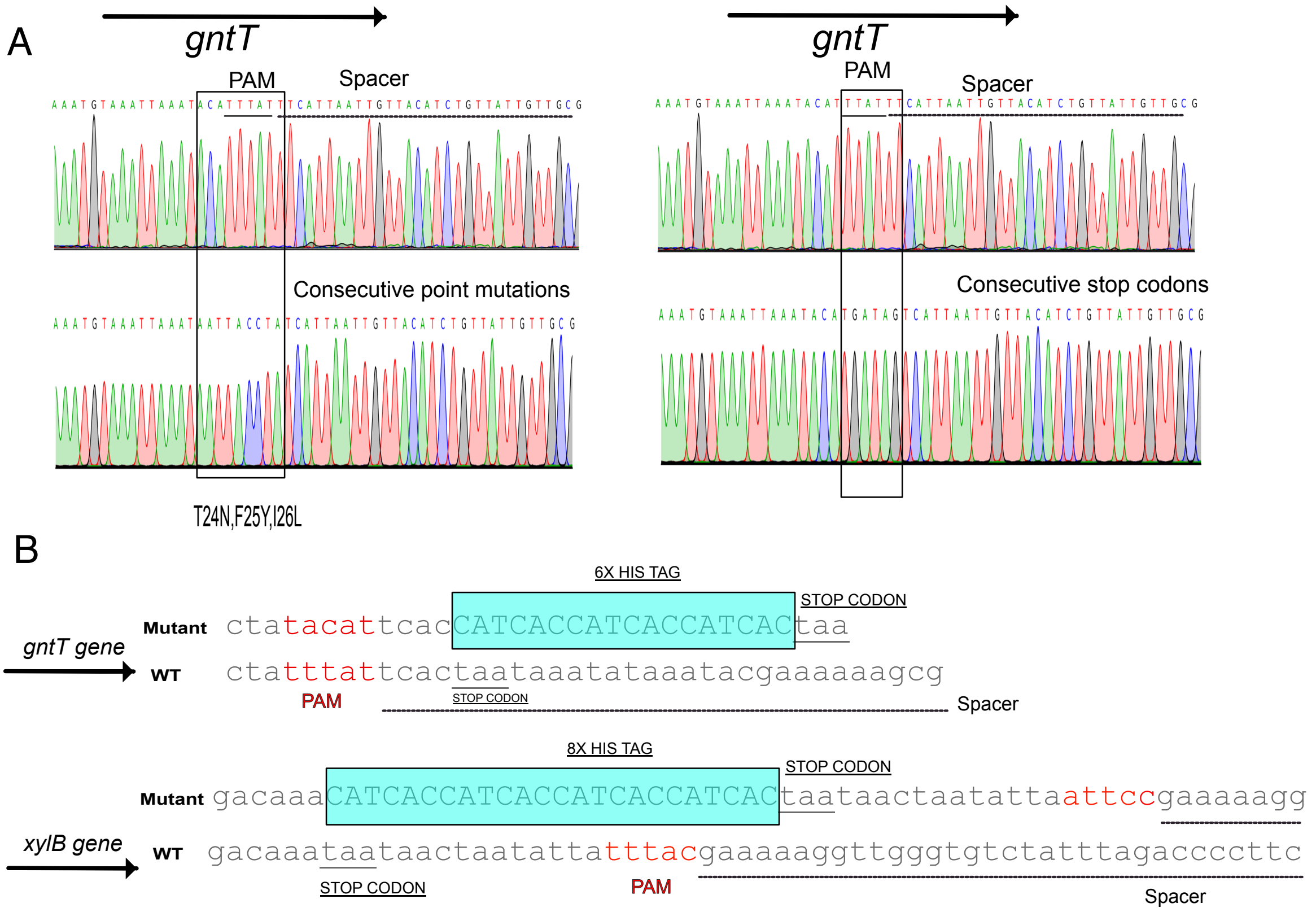

Fig S7: A) Chromatograms of CRISPR-guided introduction of consecutive stop codons and site-directed point mutations within the *gntT* locus using phosphorothioate-modified single-stranded oligonucleotides. WT genomic sequences and corresponding edited alleles are shown, with PAM sites and protospacers indicated. Insertion of consecutive stop codons resulted in premature termination of *gntT*, while targeted nucleotide substitutions generated the T24N, F25Y, and I26L amino-acid replacements. B) WT and mutant sequences showing CRISPR-assisted C-terminal tagging edits done on *gntT* and *xylB* gene. A 6×His tag was inserted at the end of the *gntT* gene (before the stop codon), whereas the *xylB* locus was tagged with an 8×His sequence. Spacer-PAM positions are indicated relative to each open reading frame.

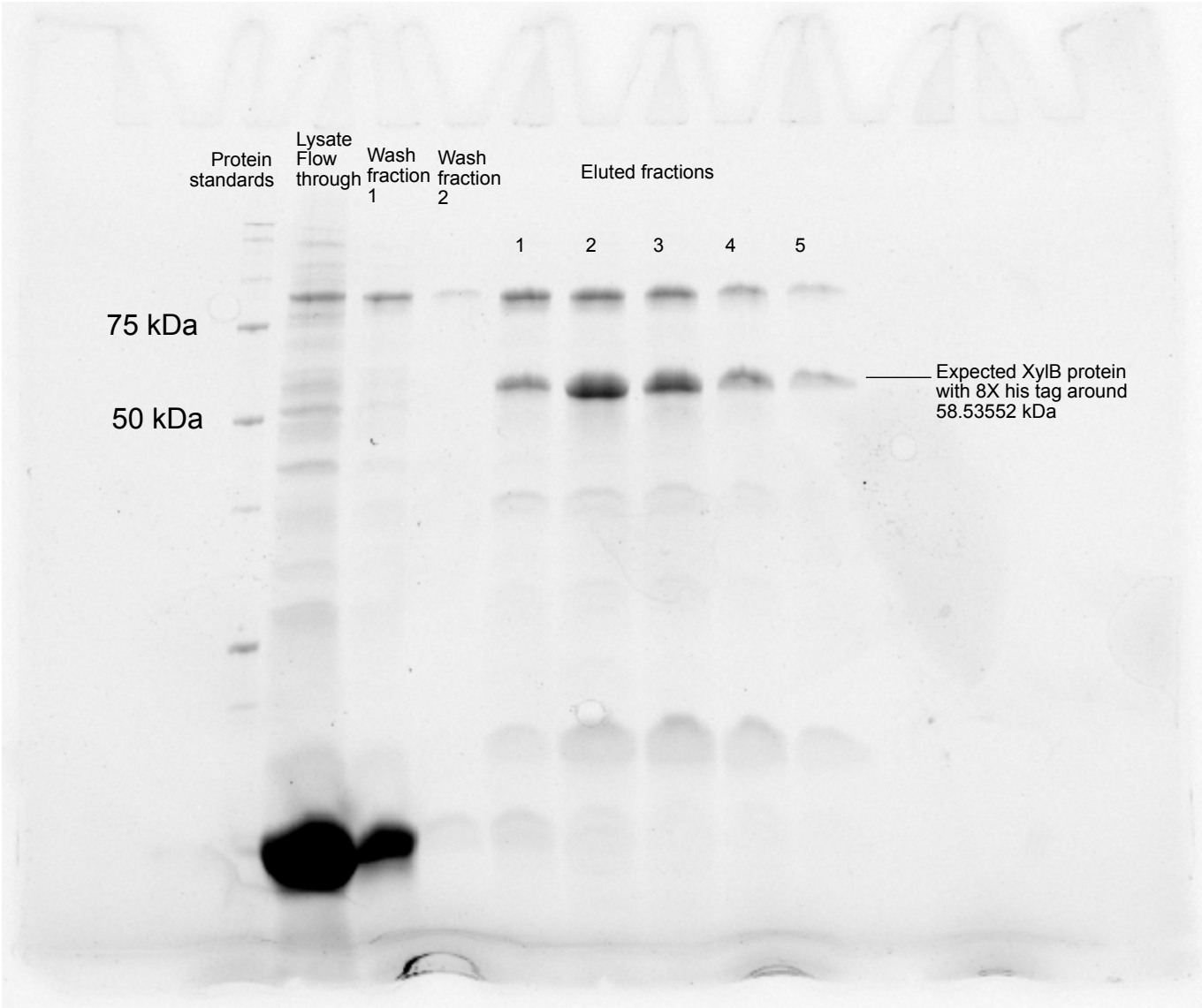

Fig S8: Ni-NTA affinity purification of C-terminal His-tagged XylB protein. SDS-PAGE analysis showing expected band (~58.5 kDa) in elution fractions, confirming successful purification of 8×His-tagged XylB.
